# Supplementary material for: Genomic and Experimental Investigations of Auriscalpium and Strobilurus Fungi Reveal New Insights into Pinecone Decomposition
Source: J Fungi (Basel). 2021 Aug 23;7(8):679. doi: 10.3390/jof7080679 (PMC8401616; doi:10.3390/jof7080679)
Supplement: Supplementary file 1 [file jof-07-00679-s001.zip › jof-1317844-supplementary/Supplementary Materials/Fig. S1.pdf]

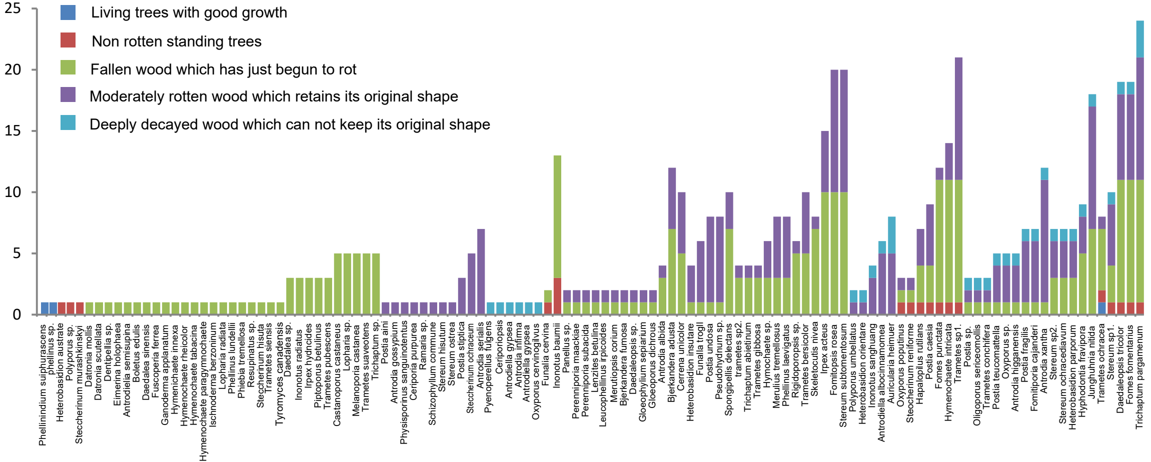

Living trees with good growth
Non rotten standing trees
Fallen wood which has just begun to rot
Moderately rotten wood which retains its original shape
Deeply decayed wood which can not keep its original shape
0
5
10
15
20
25
Phellinindium sulphurascens
phellinus sp.
Heterobasidion australe
Polyporus sp.
Steccherinum murashkinskyi
Datronia mollis
Datronia scutellata
Dentipellia sp.
Eimerina holophaea
Antrodiella semisupina
Boletus edulis
Daedalea sinensis
Fuscoporia ferrea
Ganoderma applanatum
Hymenichaete innexa
Hymenochaete rheicolor
Hymenochaete tabacina
Hymenochaete paragymnochaete
Ischnoderma berzoninum
Lopharia radiata
Phellinus lundellii
Phlebia tremellosa
Resupinatus sp.
Steccherinum hisuta
Trametes siensis
Tyromyces canadensis
Daedalea sp.
Inonotus radiatus
Irpex hydncides
Piptoporus betulinus
Trametes pubescens
Castanoporus castaneus
Lopharia sp.
Melanoporia castanea
Trametes suaveotens
Trichaptum sp.
Postia aini
Antrodia gossypium
Physisporinus sanguinotentus
Ceriporia purpurea
Ramaria sp.
Schizophyllum commune
Stereum hisutum
Stereum ostrea
Postia stiptica
Steccherinum ochraceum
Antrodia serialis
Pyenoperellus fulgens
Ceriporiopsis
Antrodiella gypsea
Antrodia infirma
Antrodiella gypsea
Oxyporus cervinogilvus
Funalia cervina
Inonotus baumii
Panellus sp.
Perenniporia maackiae
Perenniporia subacida
Lenzites betulina
Leucophellinus irpicoides
Merutiopsis corium
Bjerkandera fumosa
Daedaleopsis sp.
Gloeophyllum sepiarium
Gloeoporus dichrous
Antrodia albida
Bjerkandera adusta
Cerrena unicolor
Heterobasidion insutare
Funalia trogil
Postia undosa
Pseudohydnum sp.
Spongipellis delectans
trametes sp2.
Trichaptum abietinum
Trametes gibbosa
Hymochaete sp.
Merulius tremellosus
Phellinus laevigatus
Rigidoporopsis sp.
Trametes bersicolor
Skeletocutis nivea
Irpex acteus
Fomitopsis rosea
Stereum subtomentosum
Polyporus umbellatus
Heterobasidion orientare
Inonotus sanghuang
Antrodiella albocinnamomea
Auricularia heimuer
Oxyporus populinus
Steccherinum reniforme
Hapalopilus rutilans
Postia caesia
Fomes punctata
Hymenochaete intricata
Trametes sp1.
Postia sp.
Oligoporus sericeomollis
Trametes conchifera
Postia leucomattella
Oxyporus sp.
Antrodia higganensis
Postia fragilis
Fomitiporia cajanderi
Antrodia xantha
Stereum sp2.
Stereum ochracediavum
Heterobasidion parporum
Hyphodontia fravipora
Junghuhnia nitida
Trametes ochracea
Stereum sp1.
Daedaleopsis tricolor
Fomes fomentarius
Trichaptum pargamenum
