# Supplementary material for: Genomic and Experimental Investigations of Auriscalpium and Strobilurus Fungi Reveal New Insights into Pinecone Decomposition
Source: J Fungi (Basel). 2021 Aug 23;7(8):679. doi: 10.3390/jof7080679 (PMC8401616; doi:10.3390/jof7080679)
Supplement: Supplementary file 1 [file jof-07-00679-s001.zip › jof-1317844-supplementary/Supplementary Materials/Supplementary Material.pdf]

## Supplemental Information for:

Article title: Genomic and Experimental Investigations of *Auriscalpium* and *Strobilurus* Fungi Reveal New Insights into Pinecone Decomposition

Authors: Pan-Meng Wang1,2, 3, Jian-Ping Xu4, Gang Wu1,2, Tie-Zhi Liu5, Zhu L. Yang1,2*

**Figure S1** The distribution characteristics of wood decay fungi in different degrees of decay in Fenglin Nature Reserve (data from Zhang and Wei 2016). For the graphs, the x-axis shows different species and the y-axis shows number of occurrences of collected or observed fruiting bodies on wood in different years in Fenglin Nature Reserve. Different colors represent different levels of decay, and lengths represent numbers.

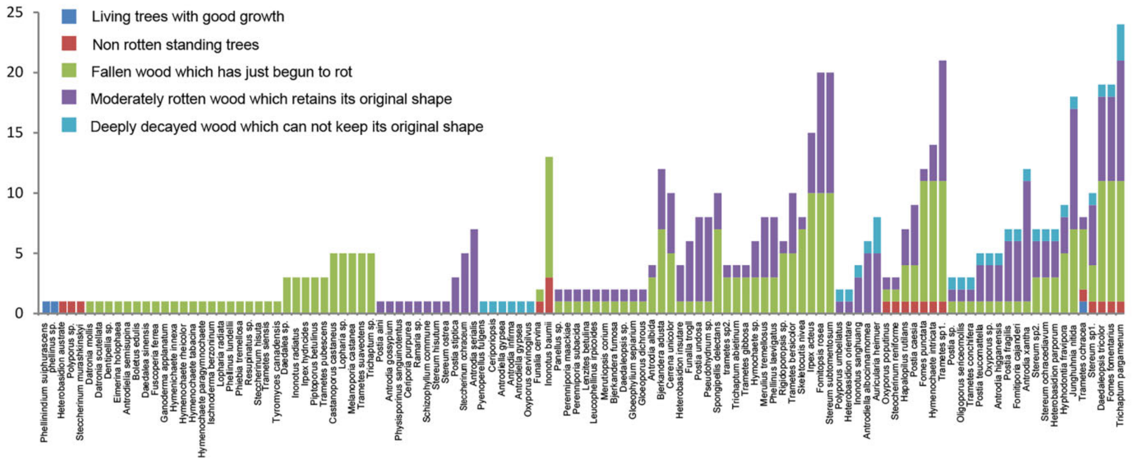

**Figure S2** The successive decomposition of *Pinus armandii* cones by *Auriscalpium* and *Strobilurus.*
(a–b) *A. microsporum* fruiting from 2017 to 2019. (c–d) *S. pachcystidiatus* fruiting from 2018 to 2020. (e–f) *S. orientalis* fruiting from 2019 to now.

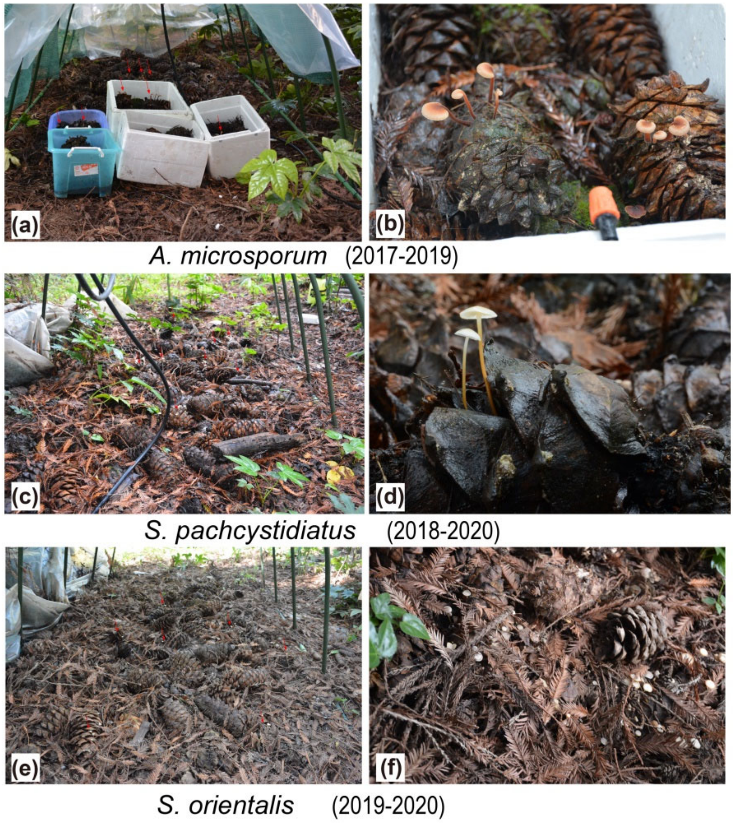
(a)
(b)
A. microsporum (2017-2019)
(c)
(d)
S. pachcystidiatus (2018-2020)
(e)
(f)
S. orientalis (2019-2020)

Figure S3: The successive decomposition of cones by *Auriscalpium* and *Strobilurus* in the field. (a) *Auriscalpium* (white arrow) and *Strobilurus* (red arrow) on cone of *P. armandii*. (b-f) *Auriscalpium* (white arrow) and *Strobilurus* (red arrow) on cone of *P*. subgenus *Pinus*.

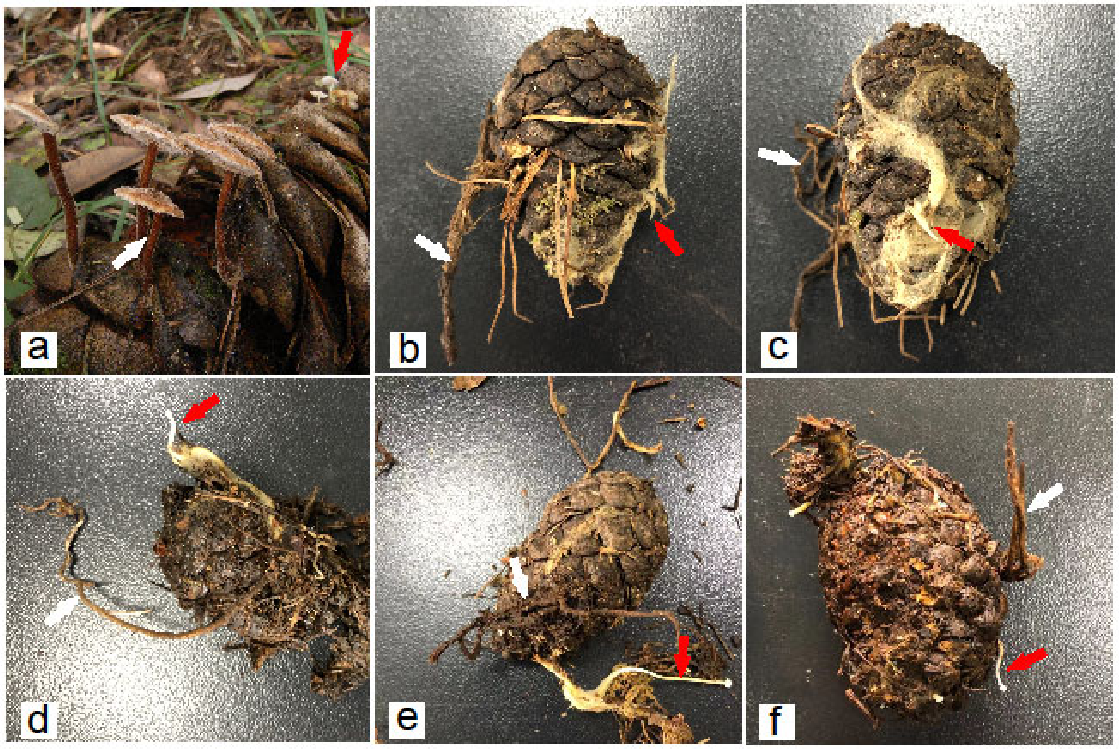


**Figure S4** The abundance and distributions of lignocellulolytic genes of *Auriscalpium* and *Strobilurus* demonstrated by multidimensional clustering approaches. (a) wood decay fungi (WDF) plotted on the first two principal components from principal

component analyses (PCA) of lignocellulolytic genes. (b) Heatmap analysis of lignocellulolytic genes Numbers of family members in each genome are shown. Overrepresented (+4 to 0) and underrepresented (0 to −4) numbers are depicted as scores for each line in heatmap. The clustering on the left involves gene families with the same pattern in number. On the right is the name of the gene family.

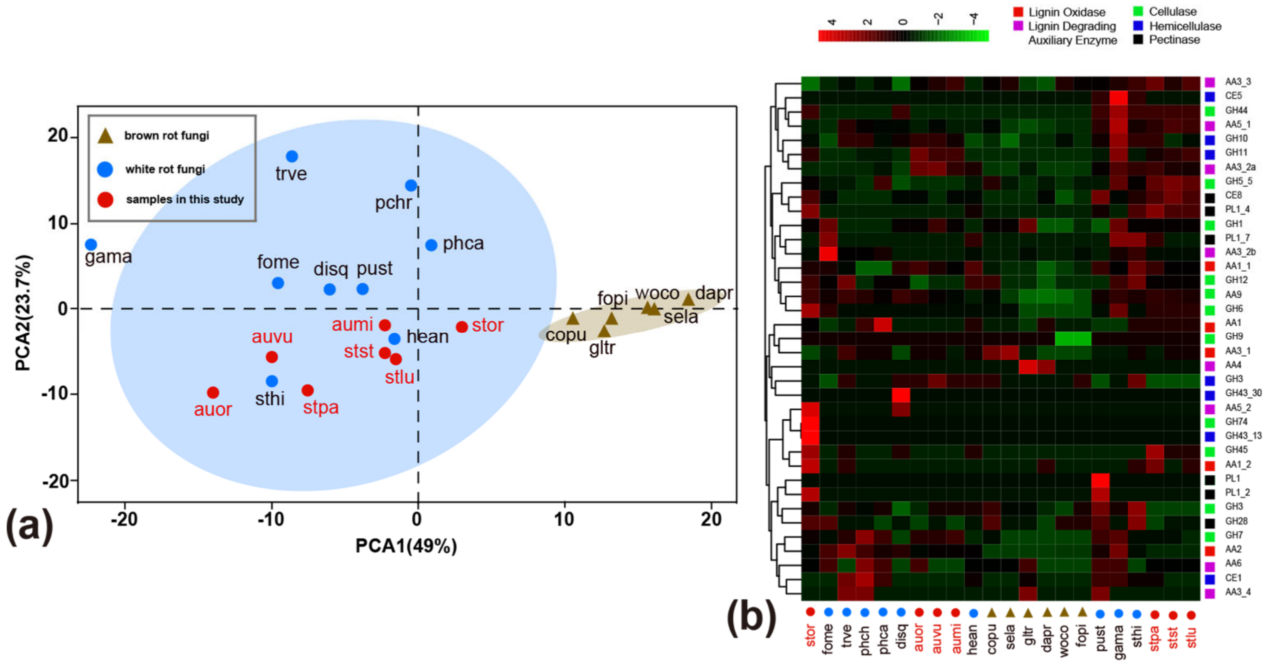


**Figure S5** Analyses of carbohydrate-active enzymes (CAZymes) and lignocellulolytic genes within genera of *Auriscalpium* and *Strobilurus* derived from heatmap, and principal component analyses (PCA). (a) Heatmap analysis of three fungi of CAZymes in

*Auriscalpium*. (b) Heatmap analysis of four fungi of CAZymes in *Strobilurus*. (c) Heatmap analysis of three fungi of lignocellulolytic genes in *Auriscalpium*. (d) Heatmap analysis of four fungi of lignocellulolytic genes in *Strobilurus*.

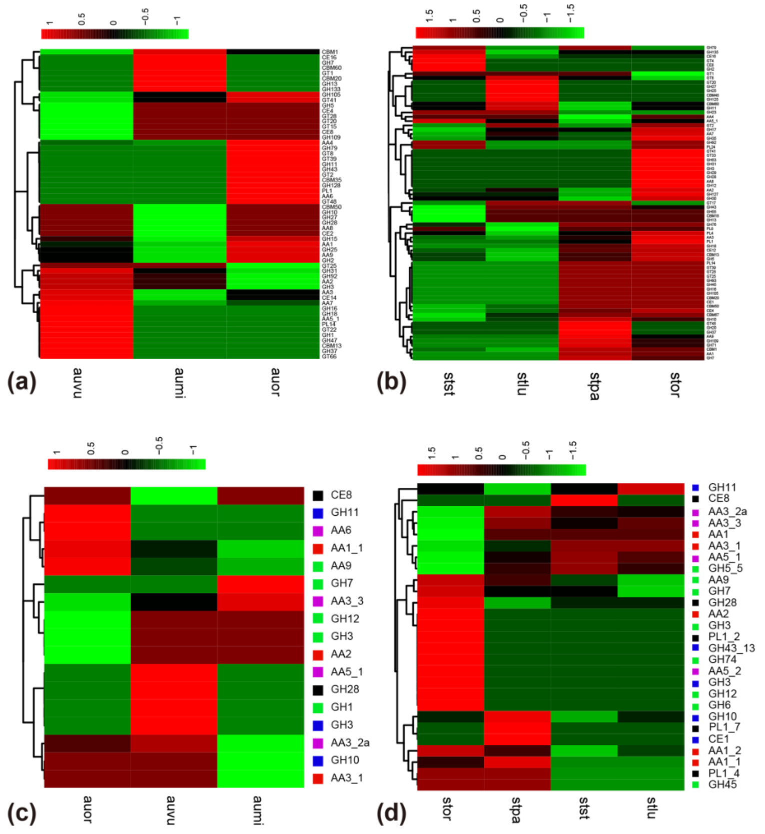


**Table S1. The collection information of fungi in *Auriscalpium* and *Strobilurus*.**

This file is too big. We uploaded it separately.

**Table S2.** Sequencing statistics.

| Library No. | Sequencing Platform | Insert size (bp) | No. Clean Reads | Sizes (M) | Genome sizes (M) | Coverage (X) |
|---|---|---|---|---|---|---|
| *S. pachycystidiatus* | Illumina Hiseq 2000 | 150 | 35114772 | 5236 | 51.82 | 101.04 |
| *S. pachycystidiatus* | Pacbio sequel | N50 18794 | 594913 | 9602 | 51.82 | 185.30 |
| *S. stephanocystis* | Illumina Hiseq 2000 | 150 | 4171448 | 6252 | 42.38 | 147.52 |
| *S. stephanocystis* | Pacbio sequel | N50 18254 | 1117580 | 16362 | 42.38 | 386.08 |
| *S. luchuensis* | Illumina Hiseq 2000 | 150 | 64564120 | 9662 | 46.71 | 206.85 |
| *S. luchuensis* | Pacbio sequel | N50 20259 | 427804 | 5965 | 46.71 | 127.70 |
| *S. orientalis* | Illumina Hiseq 2000 | 150 | 60955718 | 9143 | 51.25 | 178.4 |
| *S. orientalis* | Pacbio sequel | N50 20128 | 787474 | 11012 | 51.25 | 214.87 |
| *A. vulgare* | Illumina Hiseq 2000 | 150 | 40359420 | 6048 | 51.68 | 117.03 |
| *A. vulgare* | Pacbio sequel | N50 19574 | 1090374 | 16111 | 51.68 | 311.75 |
| *A. microsporum* | Illumina Hiseq 2000 | 150 | 38576496 | 5784 | 43.46 | 133.09 |
| *A. microsporum* | Pacbio sequel | N50 15594 | 445797 | 5004 | 43.46 | 115.14 |

| | | | | | | |
|---|---|---|---|---|---|---|
| *A. orientale* | Illumina Hiseq 2000 | 150 | 43129912 | 6468 | 45.40 | 142.47 |
| *A. orientale* | Pacbio sequel | N50 15903 | 340067 | 3897 | 45.40 | 85.84 |

**Table S3.** Assessment of the protein gene set completeness in *Auriscalpium* and *Strobilurus* using BUSCO.

| Busco | *A. vulgare* | *A. microsporum* | *A. orientale* | *S. stephanocystis* | *S. pachycystidiatus* | *S. luchuensis* | *S. orientalis* |
|---|---|---|---|---|---|---|---|
| Complete BUSCOs (C) | 274 (92.4 %) | 286 (98.6 %) | 282 (97.2 %) | 284 (98.0 %) | 290 (100.0 %) | 287 (98.9 %) | 286 (98.6%) |
| Complete and single-copy BUSCOs (S) | 243 (86.2%) | 271 (93.4 %) | 275 (94.8 %) | 276 (95.2 %) | 277 (95.5 %) | 277 (95.5 %) | 273 (94.1%) |
| Complete and duplicated BUSCOs (D) | 31 (6.2 %) | 15 (5.2 %) | 7 (2.4 %) | 8 (2.8 %) | 13 (4.5 %) | 10 (3.4 %) | 13(4.5%) |
| Fragmented BUSCOs (F) | 9 (4.8 %) | 3 (1.0 %) | 6 (2.1 %) | 2 (0.7 %) | 0 (0.0 %) | 2 (0.7 %) | 2 (0.7 %) |
| Missing BUSCOs (M) | 7 (2.8 %) | 1 (0.4 %) | 2 (0.7 %) | 4 (1.3 %) | 0 (0.0 %) | 1 (0.4 %) | 2 (0.7 %) |
| Total BUSCO groups searched | 290 (100%) | 290 (100%) | 290 (100%) | 290 (100%) | 290 (100%) | 290 (100%) | 290 (100%) |

**Table S4.** Gene distribution of carbohydrate-active enzymes (CAZymes) in *Auriscalpium* and *Strobilurus* fungi and the other 15 fungi.

| gene | dapr | fopi | copu | gltr | sela | woco | phca | phch | hean | sthi | auvu | aumi | auor | stst | stlu | stpa | stor | gama | disq | trve | fome | pust |
|---|---|---|---|---|---|---|---|---|---|---|---|---|---|---|---|---|---|---|---|---|---|---|

| | | | | | | | | | | | | | | | | | | | | | | |
|---|---|---|---|---|---|---|---|---|---|---|---|---|---|---|---|---|---|---|---|---|---|---|
| AA1 | 5 | 7 | 8 | 5 | 6 | 5 | 10 | 5 | 18 | 21 | 12 | 10 | 15 | 13 | 13 | 18 | 17 | 9 | 13 | 10 | 11 | 13 |
| AA10 | 0 | 0 | 0 | 0 | 0 | 0 | 0 | 0 | 0 | 0 | 0 | 0 | 0 | 0 | 0 | 0 | 0 | 1 | 0 | 0 | 0 | 0 |
| AA12 | 0 | 0 | 0 | 0 | 0 | 0 | 3 | 1 | 0 | 0 | 0 | 0 | 0 | 0 | 0 | 0 | 0 | 1 | 0 | 0 | 0 | 1 |
| AA2 | 1 | 2 | 1 | 1 | 1 | 2 | 12 | 17 | 13 | 8 | 11 | 10 | 8 | 5 | 6 | 4 | 8 | 32 | 14 | 27 | 18 | 13 |
| AA3 | 9 | 21 | 26 | 24 | 17 | 15 | 59 | 39 | 37 | 52 | 51 | 39 | 45 | 42 | 41 | 45 | 49 | 43 | 36 | 25 | 31 | 26 |
| AA4 | 4 | 3 | 5 | 7 | 5 | 4 | 6 | 4 | 4 | 4 | 4 | 3 | 13 | 5 | 5 | 3 | 5 | 4 | 3 | 4 | 4 | 2 |
| AA5 | 3 | 4 | 6 | 2 | 3 | 4 | 6 | 7 | 5 | 8 | 6 | 5 | 5 | 11 | 10 | 9 | 10 | 17 | 9 | 9 | 4 | 9 |
| AA6 | 1 | 1 | 2 | 3 | 2 | 1 | 3 | 4 | 2 | 1 | 1 | 1 | 3 | 2 | 2 | 2 | 2 | 3 | 1 | 1 | 3 | 3 |
| AA7 | 5 | 12 | 9 | 13 | 8 | 12 | 20 | 10 | 18 | 15 | 30 | 21 | 23 | 22 | 27 | 24 | 31 | 30 | 11 | 9 | 12 | 19 |
| AA8 | 0 | 0 | 4 | 1 | 3 | 0 | 2 | 2 | 2 | 2 | 2 | 1 | 2 | 1 | 1 | 1 | 2 | 1 | 2 | 2 | 1 | 1 |
| AA9 | 0 | 4 | 10 | 4 | 5 | 2 | 11 | 16 | 10 | 16 | 15 | 13 | 17 | 16 | 16 | 19 | 17 | 19 | 16 | 18 | 13 | 14 |
| CBM1 | 1 | 0 | 2 | 1 | 7 | 0 | 27 | 32 | 16 | 17 | 18 | 30 | 24 | 22 | 21 | 26 | 25 | 49 | 18 | 22 | 6 | 21 |
| CBM12 | 0 | 0 | 0 | 0 | 0 | 0 | 0 | 0 | 0 | 3 | 0 | 0 | 0 | 0 | 0 | 0 | 0 | 0 | 0 | 0 | 0 | 0 |
| CBM13 | 1 | 9 | 1 | 1 | 3 | 0 | 0 | 2 | 1 | 1 | 4 | 1 | 1 | 4 | 3 | 5 | 6 | 12 | 8 | 0 | 1 | 4 |
| CBM18 | 1 | 1 | 0 | 0 | 1 | 1 | 0 | 0 | 1 | 1 | 1 | 1 | 1 | 2 | 4 | 4 | 4 | 2 | 1 | 1 | 0 | 1 |
| CBM20 | 1 | 2 | 2 | 2 | 2 | 1 | 3 | 2 | 3 | 5 | 3 | 5 | 3 | 3 | 3 | 4 | 4 | 3 | 2 | 4 | 2 | 4 |

| | | | | | | | | | | | | | | | | | | | | | |
|---|---|---|---|---|---|---|---|---|---|---|---|---|---|---|---|---|---|---|---|---|---|
| CBM21 | 3 | 2 | 2 | 2 | 1 | 1 | 2 | 2 | 2 | 1 | 3 | 3 | 3 | 4 | 4 | 4 | 4 | 1 | 2 | 2 | 2 | 3 |
| CBM32 | 0 | 0 | 0 | 0 | 0 | 0 | 0 | 0 | 0 | 1 | 0 | 0 | 0 | 0 | 0 | 0 | 0 | 0 | 0 | 0 | 0 | 0 |
| CBM35 | 1 | 1 | 0 | 2 | 1 | 1 | 1 | 1 | 2 | 1 | 1 | 1 | 2 | 1 | 1 | 1 | 1 | 1 | 1 | 1 | 1 | 1 |
| CBM38 | 0 | 0 | 1 | 0 | 0 | 0 | 0 | 0 | 0 | 0 | 0 | 0 | 0 | 0 | 0 | 0 | 0 | 0 | 0 | 0 | 0 | 0 |
| CBM42 | 0 | 0 | 0 | 0 | 0 | 0 | 0 | 0 | 0 | 0 | 0 | 0 | 0 | 0 | 0 | 0 | 0 | 7 | 0 | 0 | 0 | 2 |
| CBM43 | 1 | 1 | 1 | 1 | 2 | 1 | 1 | 1 | 1 | 2 | 1 | 1 | 1 | 1 | 1 | 1 | 1 | 1 | 1 | 1 | 1 | 1 |
| CBM48 | 1 | 1 | 1 | 1 | 2 | 1 | 2 | 2 | 1 | 1 | 1 | 1 | 1 | 1 | 2 | 1 | 1 | 1 | 2 | 1 | 1 | 2 |
| CBM5 | 1 | 0 | 0 | 0 | 0 | 0 | 0 | 0 | 0 | 7 | 0 | 0 | 0 | 0 | 0 | 0 | 0 | 0 | 0 | 0 | 0 | 0 |
| CBM50 | 2 | 6 | 2 | 3 | 8 | 3 | 5 | 7 | 5 | 4 | 8 | 7 | 8 | 4 | 5 | 8 | 8 | 5 | 4 | 6 | 6 | 6 |
| CBM60 | 0 | 0 | 0 | 0 | 0 | 0 | 0 | 0 | 0 | 1 | 3 | 4 | 3 | 3 | 4 | 2 | 3 | 6 | 0 | 0 | 0 | 0 |
| CBM63 | 0 | 0 | 0 | 0 | 0 | 0 | 0 | 0 | 0 | 0 | 0 | 0 | 0 | 1 | 1 | 1 | 1 | 0 | 0 | 0 | 0 | 0 |
| CBM67 | 0 | 0 | 0 | 0 | 0 | 0 | 0 | 0 | 0 | 0 | 0 | 0 | 0 | 3 | 4 | 5 | 5 | 9 | 0 | 0 | 0 | 0 |
| CE1 | 0 | 0 | 0 | 2 | 0 | 0 | 2 | 4 | 2 | 1 | 0 | 0 | 0 | 0 | 0 | 1 | 1 | 2 | 0 | 3 | 0 | 2 |
| CE12 | 0 | 0 | 0 | 0 | 0 | 0 | 0 | 0 | 2 | 3 | 0 | 0 | 0 | 3 | 2 | 5 | 6 | 4 | 2 | 0 | 2 | 1 |
| CE14 | 1 | 1 | 0 | 1 | 1 | 1 | 1 | 1 | 1 | 0 | 2 | 0 | 1 | 0 | 0 | 0 | 0 | 1 | 0 | 1 | 0 | 1 |
| CE15 | 1 | 1 | 0 | 1 | 0 | 1 | 3 | 2 | 1 | 1 | 1 | 1 | 1 | 1 | 1 | 1 | 1 | 1 | 2 | 2 | 1 | 2 |

| | | | | | | | | | | | | | | | | | | | | | | |
|---|---|---|---|---|---|---|---|---|---|---|---|---|---|---|---|---|---|---|---|---|---|---|
| CE16 | 4 | 9 | 8 | 5 | 4 | 5 | 5 | 7 | 7 | 11 | 6 | 7 | 6 | 18 | 14 | 15 | 15 | 10 | 13 | 8 | 6 | 11 |
| CE2 | 1 | 1 | 1 | 1 | 1 | 0 | 0 | 1 | 1 | 1 | 1 | 0 | 1 | 1 | 1 | 1 | 1 | 2 | 1 | 1 | 1 | 2 |
| CE4 | 14 | 5 | 11 | 8 | 8 | 5 | 4 | 6 | 7 | 9 | 5 | 6 | 6 | 9 | 10 | 13 | 14 | 14 | 7 | 5 | 7 | 7 |
| CE5 | 0 | 0 | 1 | 0 | 0 | 0 | 0 | 0 | 0 | 1 | 0 | 0 | 0 | 0 | 0 | 0 | 0 | 6 | 0 | 0 | 0 | 1 |
| CE8 | 3 | 2 | 2 | 2 | 2 | 1 | 2 | 2 | 3 | 4 | 2 | 3 | 3 | 6 | 5 | 5 | 5 | 3 | 3 | 2 | 3 | 6 |
| CE9 | 0 | 1 | 1 | 1 | 1 | 1 | 1 | 1 | 3 | 1 | 1 | 1 | 1 | 1 | 1 | 1 | 1 | 1 | 1 | 1 | 1 | 1 |
| GH1 | 1 | 2 | 3 | 5 | 3 | 1 | 2 | 2 | 2 | 3 | 4 | 3 | 3 | 3 | 3 | 3 | 3 | 5 | 3 | 2 | 5 | 1 |
| GH10 | 3 | 3 | 3 | 3 | 1 | 4 | 5 | 6 | 2 | 6 | 6 | 5 | 6 | 3 | 4 | 6 | 5 | 9 | 5 | 6 | 4 | 5 |
| GH105 | 2 | 4 | 2 | 6 | 4 | 3 | 4 | 4 | 5 | 4 | 2 | 3 | 4 | 7 | 7 | 8 | 8 | 6 | 4 | 3 | 4 | 7 |
| GH109 | 4 | 5 | 1 | 4 | 3 | 4 | 4 | 4 | 3 | 4 | 3 | 4 | 4 | 1 | 1 | 3 | 2 | 5 | 3 | 5 | 2 | 5 |
| GH11 | 0 | 0 | 0 | 0 | 0 | 0 | 1 | 1 | 0 | 1 | 5 | 5 | 7 | 4 | 5 | 3 | 4 | 8 | 0 | 0 | 0 | 1 |
| GH114 | 0 | 0 | 1 | 0 | 0 | 0 | 0 | 0 | 0 | 0 | 0 | 0 | 0 | 0 | 0 | 0 | 0 | 0 | 0 | 0 | 0 | 0 |
| GH115 | 2 | 1 | 2 | 2 | 1 | 2 | 1 | 1 | 1 | 2 | 1 | 1 | 1 | 1 | 1 | 1 | 1 | 1 | 2 | 2 | 3 | 1 |
| GH12 | 1 | 2 | 4 | 2 | 2 | 2 | 3 | 2 | 4 | 5 | 2 | 2 | 2 | 3 | 3 | 3 | 4 | 4 | 3 | 5 | 3 | 2 |
| GH125 | 0 | 1 | 1 | 1 | 1 | 1 | 1 | 1 | 1 | 1 | 1 | 1 | 1 | 1 | 2 | 1 | 1 | 1 | 1 | 1 | 1 | 1 |
| GH127 | 1 | 2 | 2 | 2 | 1 | 2 | 1 | 1 | 1 | 1 | 0 | 0 | 0 | 2 | 2 | 1 | 4 | 2 | 1 | 3 | 1 | 3 |

| | | | | | | | | | | | | | | | | | | | | | | |
|---|---|---|---|---|---|---|---|---|---|---|---|---|---|---|---|---|---|---|---|---|---|---|
| GH128 | 2 | 4 | 9 | 6 | 5 | 2 | 4 | 4 | 1 | 6 | 3 | 3 | 5 | 7 | 7 | 7 | 7 | 6 | 5 | 4 | 3 | 3 |
| GH13 | 11 | 7 | 6 | 9 | 7 | 10 | 10 | 9 | 8 | 14 | 7 | 10 | 7 | 13 | 14 | 14 | 14 | 9 | 8 | 7 | 6 | 10 |
| GH131 | 1 | 1 | 2 | 1 | 2 | 0 | 2 | 3 | 2 | 3 | 2 | 2 | 2 | 2 | 2 | 2 | 2 | 4 | 3 | 3 | 2 | 2 |
| GH133 | 1 | 1 | 1 | 1 | 1 | 1 | 1 | 1 | 1 | 1 | 1 | 3 | 1 | 1 | 1 | 1 | 1 | 1 | 1 | 1 | 1 | 1 |
| GH134 | 0 | 0 | 0 | 0 | 0 | 0 | 0 | 0 | 0 | 0 | 0 | 0 | 0 | 0 | 0 | 0 | 0 | 1 | 0 | 0 | 0 | 0 |
| GH135 | 0 | 0 | 0 | 0 | 0 | 0 | 1 | 1 | 0 | 1 | 0 | 0 | 0 | 3 | 1 | 2 | 2 | 1 | 2 | 2 | 0 | 0 |
| GH140 | 0 | 0 | 0 | 0 | 0 | 0 | 0 | 0 | 2 | 1 | 0 | 0 | 0 | 0 | 0 | 0 | 0 | 0 | 0 | 0 | 0 | 0 |
| GH145 | 0 | 0 | 1 | 1 | 1 | 0 | 1 | 1 | 1 | 1 | 1 | 1 | 1 | 1 | 1 | 1 | 1 | 1 | 1 | 1 | 1 | 1 |
| GH15 | 2 | 3 | 2 | 2 | 2 | 2 | 2 | 2 | 4 | 3 | 10 | 8 | 11 | 3 | 3 | 3 | 3 | 3 | 2 | 4 | 1 | 4 |
| GH16 | 13 | 23 | 23 | 28 | 19 | 19 | 24 | 22 | 21 | 26 | 23 | 21 | 21 | 32 | 32 | 33 | 33 | 23 | 34 | 29 | 28 | 26 |
| GH17 | 3 | 1 | 1 | 1 | 1 | 1 | 1 | 1 | 1 | 1 | 1 | 1 | 1 | 3 | 4 | 4 | 5 | 4 | 1 | 1 | 4 | 1 |
| GH18 | 7 | 18 | 28 | 11 | 11 | 11 | 11 | 11 | 9 | 21 | 14 | 13 | 13 | 18 | 18 | 20 | 21 | 19 | 22 | 20 | 16 | 13 |
| GH2 | 3 | 4 | 5 | 4 | 2 | 3 | 2 | 2 | 3 | 3 | 5 | 4 | 6 | 3 | 2 | 2 | 2 | 3 | 4 | 5 | 2 | 4 |
| GH20 | 3 | 4 | 4 | 4 | 2 | 2 | 3 | 4 | 4 | 4 | 2 | 2 | 2 | 2 | 2 | 3 | 2 | 2 | 4 | 5 | 6 | 6 |
| GH23 | 2 | 1 | 1 | 1 | 1 | 1 | 1 | 1 | 1 | 1 | 1 | 1 | 1 | 1 | 1 | 0 | 0 | 1 | 1 | 1 | 1 | 1 |
| GH24 | 0 | 0 | 0 | 0 | 0 | 0 | 0 | 0 | 0 | 0 | 0 | 0 | 0 | 0 | 0 | 0 | 0 | 2 | 0 | 0 | 0 | 0 |

| | | | | | | | | | | | | | | | | | | | | | | |
|---|---|---|---|---|---|---|---|---|---|---|---|---|---|---|---|---|---|---|---|---|---|---|
| GH25 | 0 | 0 | 2 | 2 | 1 | 0 | 0 | 1 | 0 | 0 | 1 | 0 | 2 | 1 | 2 | 1 | 1 | 1 | 2 | 1 | 1 | 1 |
| GH26 | 4 | 0 | 0 | 0 | 0 | 0 | 0 | 0 | 0 | 0 | 0 | 0 | 0 | 0 | 0 | 0 | 0 | 0 | 0 | 0 | 0 | 0 |
| GH27 | 1 | 4 | 4 | 3 | 3 | 3 | 3 | 3 | 4 | 5 | 3 | 2 | 3 | 5 | 6 | 5 | 5 | 8 | 6 | 4 | 4 | 5 |
| GH28 | 6 | 12 | 13 | 10 | 7 | 9 | 4 | 5 | 8 | 17 | 3 | 2 | 3 | 7 | 7 | 7 | 9 | 18 | 7 | 10 | 16 | 13 |
| GH29 | 2 | 0 | 4 | 1 | 1 | 0 | 0 | 0 | 2 | 4 | 0 | 0 | 0 | 1 | 1 | 1 | 2 | 1 | 0 | 0 | 0 | 1 |
| GH3 | 9 | 11 | 13 | 10 | 10 | 8 | 11 | 10 | 12 | 17 | 13 | 12 | 10 | 7 | 7 | 7 | 11 | 11 | 8 | 12 | 8 | 13 |
| GH30 | 4 | 7 | 7 | 3 | 2 | 2 | 3 | 2 | 2 | 5 | 3 | 3 | 3 | 3 | 3 | 2 | 4 | 2 | 2 | 4 | 2 | 3 |
| GH31 | 6 | 4 | 11 | 5 | 5 | 5 | 6 | 6 | 9 | 8 | 8 | 7 | 6 | 4 | 4 | 4 | 5 | 4 | 6 | 5 | 5 | 7 |
| GH32 | 1 | 4 | 1 | 1 | 0 | 0 | 0 | 0 | 1 | 1 | 0 | 0 | 0 | 0 | 0 | 0 | 0 | 1 | 2 | 3 | 0 | 1 |
| GH35 | 1 | 2 | 2 | 2 | 3 | 2 | 4 | 3 | 4 | 7 | 1 | 1 | 1 | 4 | 6 | 5 | 9 | 8 | 3 | 2 | 2 | 4 |
| GH37 | 2 | 2 | 4 | 2 | 2 | 3 | 2 | 2 | 2 | 2 | 4 | 2 | 2 | 2 | 2 | 3 | 2 | 2 | 3 | 2 | 3 | 2 |
| GH38 | 1 | 1 | 1 | 1 | 1 | 1 | 2 | 1 | 1 | 1 | 1 | 1 | 1 | 1 | 1 | 1 | 1 | 1 | 1 | 1 | 1 | 1 |
| GH43 | 5 | 7 | 6 | 6 | 2 | 1 | 4 | 4 | 6 | 11 | 3 | 3 | 4 | 10 | 14 | 14 | 13 | 10 | 7 | 3 | 6 | 9 |
| GH44 | 0 | 0 | 0 | 0 | 0 | 0 | 0 | 0 | 0 | 1 | 0 | 0 | 0 | 1 | 1 | 1 | 1 | 2 | 1 | 0 | 0 | 1 |
| GH45 | 0 | 0 | 0 | 0 | 0 | 0 | 0 | 0 | 1 | 0 | 0 | 0 | 0 | 1 | 1 | 3 | 3 | 0 | 1 | 1 | 0 | 0 |
| GH47 | 4 | 5 | 9 | 4 | 5 | 5 | 5 | 5 | 6 | 6 | 7 | 5 | 5 | 8 | 8 | 8 | 8 | 10 | 5 | 5 | 5 | 5 |

| | | | | | | | | | | | | | | | | | | | | | | |
|---|---|---|---|---|---|---|---|---|---|---|---|---|---|---|---|---|---|---|---|---|---|---|
| GH5 | 24 | 19 | 20 | 19 | 20 | 18 | 23 | 19 | 16 | 20 | 18 | 19 | 19 | 26 | 25 | 27 | 28 | 37 | 20 | 22 | 19 | 17 |
| GH51 | 2 | 3 | 3 | 3 | 1 | 4 | 2 | 2 | 1 | 3 | 2 | 2 | 2 | 3 | 3 | 3 | 3 | 5 | 2 | 2 | 1 | 3 |
| GH53 | 1 | 1 | 1 | 2 | 1 | 1 | 1 | 1 | 1 | 2 | 0 | 0 | 0 | 1 | 1 | 1 | 2 | 3 | 1 | 1 | 1 | 2 |
| GH54 | 0 | 0 | 0 | 0 | 0 | 0 | 0 | 0 | 0 | 0 | 0 | 0 | 0 | 0 | 0 | 0 | 0 | 8 | 0 | 0 | 0 | 2 |
| GH55 | 2 | 3 | 4 | 2 | 5 | 3 | 2 | 2 | 1 | 2 | 2 | 2 | 2 | 3 | 4 | 4 | 4 | 3 | 2 | 2 | 1 | 3 |
| GH6 | 0 | 0 | 1 | 0 | 1 | 0 | 1 | 1 | 1 | 1 | 1 | 1 | 1 | 4 | 4 | 4 | 4 | 3 | 1 | 1 | 2 | 1 |
| GH62 | 0 | 0 | 0 | 0 | 0 | 0 | 0 | 0 | 0 | 0 | 0 | 0 | 0 | 1 | 1 | 1 | 1 | 1 | 0 | 0 | 0 | 0 |
| GH63 | 1 | 1 | 1 | 1 | 1 | 1 | 1 | 1 | 1 | 1 | 1 | 1 | 1 | 1 | 1 | 1 | 1 | 1 | 1 | 1 | 1 | 1 |
| GH7 | 0 | 0 | 2 | 0 | 0 | 0 | 5 | 8 | 1 | 3 | 4 | 5 | 4 | 5 | 5 | 8 | 7 | 8 | 3 | 4 | 2 | 5 |
| GH71 | 5 | 4 | 6 | 6 | 4 | 1 | 2 | 3 | 3 | 8 | 3 | 3 | 3 | 5 | 5 | 7 | 6 | 3 | 4 | 5 | 2 | 5 |
| GH72 | 1 | 1 | 1 | 1 | 2 | 2 | 1 | 1 | 1 | 2 | 1 | 1 | 1 | 1 | 1 | 1 | 1 | 1 | 1 | 1 | 1 | 1 |
| GH74 | 0 | 0 | 0 | 1 | 1 | 0 | 2 | 4 | 1 | 2 | 1 | 1 | 1 | 1 | 1 | 1 | 1 | 2 | 1 | 1 | 4 | 2 |
| GH75 | 0 | 0 | 0 | 0 | 0 | 0 | 0 | 0 | 0 | 1 | 0 | 0 | 0 | 0 | 0 | 0 | 0 | 0 | 0 | 0 | 0 | 0 |
| GH76 | 0 | 2 | 6 | 4 | 2 | 0 | 0 | 0 | 1 | 4 | 1 | 1 | 1 | 3 | 2 | 4 | 4 | 2 | 1 | 0 | 0 | 3 |
| GH78 | 0 | 4 | 2 | 2 | 2 | 3 | 1 | 1 | 2 | 3 | 0 | 0 | 0 | 1 | 1 | 1 | 1 | 2 | 3 | 3 | 2 | 7 |
| GH79 | 6 | 3 | 4 | 6 | 6 | 3 | 4 | 7 | 8 | 11 | 5 | 5 | 6 | 4 | 3 | 4 | 3 | 16 | 12 | 11 | 7 | 9 |

| | | | | | | | | | | | | | | | | | | | | | | |
|---|---|---|---|---|---|---|---|---|---|---|---|---|---|---|---|---|---|---|---|---|---|---|
| GH81 | 0 | 0 | 1 | 1 | 0 | 0 | 0 | 0 | 2 | 2 | 1 | 1 | 1 | 0 | 0 | 0 | 0 | 0 | 0 | 0 | 0 | 0 |
| GH85 | 2 | 1 | 1 | 1 | 1 | 1 | 1 | 1 | 1 | 1 | 1 | 1 | 1 | 1 | 1 | 1 | 1 | 1 | 1 | 1 | 1 | 1 |
| GH88 | 1 | 1 | 1 | 1 | 1 | 1 | 1 | 1 | 1 | 1 | 2 | 2 | 2 | 2 | 2 | 2 | 2 | 1 | 1 | 1 | 2 | 1 |
| GH89 | 0 | 2 | 2 | 0 | 2 | 1 | 1 | 2 | 0 | 0 | 0 | 0 | 0 | 0 | 0 | 0 | 0 | 0 | 1 | 1 | 0 | 0 |
| GH9 | 1 | 0 | 1 | 1 | 1 | 0 | 1 | 1 | 1 | 1 | 1 | 1 | 1 | 1 | 1 | 1 | 1 | 1 | 1 | 1 | 1 | 1 |
| GH92 | 1 | 5 | 4 | 4 | 3 | 3 | 4 | 4 | 3 | 6 | 7 | 6 | 4 | 5 | 4 | 4 | 5 | 7 | 5 | 4 | 4 | 4 |
| GH93 | 0 | 0 | 1 | 0 | 0 | 0 | 0 | 0 | 0 | 1 | 0 | 0 | 0 | 1 | 1 | 2 | 2 | 0 | 1 | 0 | 0 | 1 |
| GH94 | 0 | 0 | 0 | 0 | 0 | 0 | 0 | 0 | 0 | 0 | 0 | 0 | 0 | 0 | 0 | 0 | 0 | 1 | 0 | 0 | 0 | 0 |
| GH95 | 0 | 1 | 1 | 1 | 1 | 1 | 1 | 1 | 1 | 1 | 1 | 1 | 1 | 1 | 1 | 1 | 1 | 2 | 1 | 1 | 2 | 1 |
| GT1 | 14 | 5 | 3 | 2 | 2 | 3 | 8 | 6 | 2 | 5 | 2 | 3 | 2 | 6 | 6 | 6 | 5 | 13 | 8 | 20 | 10 | 12 |
| GT15 | 4 | 5 | 2 | 4 | 3 | 3 | 4 | 2 | 3 | 4 | 3 | 4 | 4 | 7 | 7 | 7 | 7 | 7 | 6 | 5 | 5 | 4 |
| GT17 | 1 | 1 | 0 | 1 | 0 | 1 | 0 | 1 | 1 | 1 | 0 | 0 | 0 | 1 | 2 | 2 | 1 | 1 | 1 | 1 | 2 | 0 |
| GT18 | 1 | 0 | 0 | 1 | 0 | 0 | 0 | 0 | 0 | 0 | 0 | 0 | 0 | 0 | 0 | 0 | 0 | 1 | 0 | 0 | 0 | 0 |
| GT2 | 8 | 13 | 9 | 10 | 14 | 10 | 10 | 11 | 9 | 12 | 9 | 9 | 14 | 10 | 11 | 10 | 11 | 12 | 10 | 12 | 7 | 10 |
| GT20 | 3 | 3 | 3 | 3 | 3 | 3 | 3 | 3 | 3 | 3 | 2 | 3 | 3 | 3 | 4 | 3 | 3 | 3 | 3 | 3 | 3 | 3 |
| GT21 | 1 | 1 | 1 | 1 | 1 | 1 | 1 | 1 | 1 | 1 | 1 | 1 | 1 | 1 | 1 | 1 | 1 | 1 | 1 | 1 | 1 | 1 |

| | | | | | | | | | | | | | | | | | | | | | | |
|---|---|---|---|---|---|---|---|---|---|---|---|---|---|---|---|---|---|---|---|---|---|---|
| GT22 | 2 | 5 | 4 | 3 | 4 | 4 | 5 | 4 | 3 | 4 | 4 | 3 | 3 | 3 | 3 | 3 | 3 | 3 | 4 | 4 | 3 | 3 |
| GT24 | 1 | 1 | 1 | 1 | 1 | 1 | 1 | 1 | 1 | 1 | 1 | 1 | 1 | 1 | 1 | 1 | 1 | 1 | 1 | 1 | 1 | 1 |
| GT25 | 1 | 1 | 3 | 1 | 1 | 1 | 0 | 1 | 1 | 1 | 1 | 1 | 0 | 2 | 2 | 3 | 3 | 1 | 0 | 0 | 0 | 1 |
| GT28 | 1 | 0 | 0 | 1 | 1 | 1 | 1 | 1 | 1 | 1 | 0 | 1 | 1 | 0 | 0 | 1 | 1 | 1 | 1 | 0 | 1 | 1 |
| GT3 | 1 | 1 | 1 | 1 | 1 | 1 | 1 | 1 | 1 | 1 | 1 | 1 | 1 | 1 | 1 | 1 | 1 | 1 | 1 | 2 | 2 | 1 |
| GT31 | 3 | 1 | 1 | 1 | 1 | 1 | 1 | 1 | 1 | 1 | 1 | 1 | 1 | 1 | 1 | 1 | 1 | 2 | 1 | 1 | 1 | 1 |
| GT32 | 5 | 1 | 1 | 2 | 1 | 1 | 1 | 1 | 1 | 1 | 1 | 1 | 1 | 1 | 1 | 1 | 1 | 1 | 1 | 1 | 2 | 1 |
| GT33 | 1 | 1 | 1 | 1 | 1 | 1 | 1 | 1 | 1 | 1 | 1 | 1 | 1 | 2 | 2 | 2 | 3 | 1 | 1 | 1 | 1 | 1 |
| GT35 | 1 | 1 | 1 | 1 | 1 | 1 | 1 | 1 | 1 | 1 | 1 | 1 | 1 | 1 | 1 | 1 | 1 | 1 | 1 | 1 | 1 | 1 |
| GT39 | 4 | 3 | 3 | 3 | 3 | 3 | 3 | 3 | 3 | 3 | 3 | 3 | 4 | 3 | 3 | 4 | 4 | 3 | 2 | 3 | 3 | 3 |
| GT4 | 6 | 4 | 3 | 5 | 4 | 4 | 5 | 4 | 4 | 4 | 4 | 4 | 4 | 6 | 5 | 5 | 5 | 5 | 4 | 4 | 3 | 6 |
| GT41 | 1 | 1 | 0 | 0 | 2 | 2 | 1 | 1 | 1 | 0 | 1 | 2 | 3 | 0 | 0 | 0 | 1 | 0 | 2 | 2 | 1 | 2 |
| GT47 | 1 | 0 | 0 | 0 | 0 | 0 | 0 | 0 | 0 | 1 | 0 | 0 | 0 | 0 | 0 | 0 | 0 | 0 | 0 | 0 | 0 | 0 |
| GT48 | 1 | 2 | 3 | 3 | 2 | 2 | 2 | 2 | 2 | 2 | 2 | 2 | 6 | 2 | 2 | 3 | 2 | 2 | 2 | 2 | 2 | 2 |
| GT49 | 1 | 1 | 1 | 1 | 1 | 1 | 1 | 1 | 1 | 1 | 1 | 1 | 1 | 1 | 1 | 1 | 1 | 1 | 1 | 1 | 1 | 1 |
| GT5 | 1 | 1 | 0 | 1 | 1 | 2 | 1 | 1 | 1 | 1 | 1 | 1 | 1 | 1 | 1 | 1 | 1 | 1 | 1 | 1 | 1 | 1 |

| | | | | | | | | | | | | | | | | | | | | | | |
|---|---|---|---|---|---|---|---|---|---|---|---|---|---|---|---|---|---|---|---|---|---|---|
| GT50 | 1 | 1 | 1 | 1 | 1 | 1 | 0 | 1 | 1 | 1 | 1 | 1 | 1 | 1 | 1 | 1 | 1 | 1 | 1 | 1 | 1 | 1 |
| GT57 | 2 | 2 | 2 | 2 | 2 | 2 | 2 | 2 | 2 | 2 | 2 | 2 | 2 | 2 | 2 | 2 | 2 | 2 | 2 | 2 | 2 | 2 |
| GT58 | 1 | 2 | 1 | 1 | 1 | 1 | 1 | 1 | 1 | 1 | 1 | 1 | 1 | 1 | 1 | 1 | 1 | 1 | 1 | 1 | 1 | 1 |
| GT59 | 1 | 1 | 1 | 1 | 1 | 1 | 1 | 1 | 1 | 1 | 1 | 1 | 1 | 1 | 1 | 1 | 1 | 1 | 1 | 1 | 1 | 1 |
| GT66 | 1 | 1 | 1 | 1 | 1 | 1 | 1 | 1 | 1 | 1 | 2 | 1 | 1 | 1 | 1 | 1 | 1 | 1 | 1 | 1 | 1 | 1 |
| GT69 | 2 | 3 | 2 | 2 | 3 | 3 | 3 | 3 | 4 | 3 | 2 | 2 | 2 | 4 | 4 | 4 | 4 | 2 | 4 | 3 | 2 | 3 |
| GT71 | 2 | 0 | 0 | 0 | 0 | 0 | 0 | 0 | 0 | 1 | 0 | 0 | 0 | 0 | 0 | 0 | 0 | 0 | 0 | 0 | 0 | 0 |
| GT76 | 1 | 1 | 1 | 1 | 1 | 1 | 1 | 1 | 1 | 1 | 1 | 1 | 1 | 1 | 1 | 1 | 1 | 1 | 1 | 1 | 1 | 1 |
| GT8 | 4 | 7 | 3 | 3 | 3 | 8 | 7 | 7 | 6 | 7 | 5 | 5 | 6 | 7 | 8 | 7 | 6 | 4 | 6 | 5 | 4 | 4 |
| GT90 | 10 | 1 | 1 | 2 | 4 | 1 | 1 | 1 | 1 | 1 | 1 | 1 | 1 | 6 | 6 | 6 | 6 | 3 | 1 | 1 | 4 | 5 |
| PL1 | 0 | 0 | 0 | 0 | 0 | 0 | 0 | 0 | 2 | 5 | 0 | 0 | 1 | 3 | 3 | 4 | 6 | 2 | 0 | 0 | 3 | 5 |
| PL10_1 | 0 | 0 | 0 | 0 | 0 | 0 | 0 | 0 | 0 | 1 | 0 | 0 | 0 | 0 | 0 | 0 | 0 | 0 | 0 | 0 | 0 | 1 |
| PL14 | 3 | 3 | 2 | 6 | 4 | 2 | 5 | 5 | 4 | 4 | 6 | 5 | 5 | 3 | 3 | 4 | 4 | 4 | 7 | 6 | 4 | 4 |
| PL24 | 0 | 0 | 0 | 0 | 0 | 0 | 0 | 0 | 0 | 0 | 0 | 0 | 0 | 1 | 0 | 0 | 1 | 1 | 0 | 0 | 0 | 0 |
| PL26 | 0 | 0 | 0 | 0 | 0 | 0 | 0 | 0 | 1 | 2 | 0 | 0 | 0 | 1 | 1 | 1 | 1 | 0 | 0 | 0 | 0 | 1 |
| PL4 | 0 | 0 | 0 | 2 | 0 | 0 | 0 | 0 | 1 | 3 | 0 | 0 | 0 | 2 | 1 | 2 | 3 | 0 | 1 | 1 | 0 | 3 |

| | | | | | | | | | | | | | | | | | | | | | | |
|---|---|---|---|---|---|---|---|---|---|---|---|---|---|---|---|---|---|---|---|---|---|---|
| PL8 | 0 | 0 | 0 | 1 | 2 | 0 | 1 | 1 | 2 | 2 | 2 | 2 | 2 | 1 | 0 | 1 | 1 | 2 | 3 | 2 | 0 | 1 |

Note: dapr: *Dacryopinax primogenitus*; copu: *Coniophora puteana*; sela: *Serpula lacrymans* ; woco: *Wolfiporia cocos*; fopi: *Fomitopsis pinicola*i; gltr: *Gloeophyllum trabeum*; gama: *Galerina marginata*; trve: *Trametes versicolor*; hean: *Heterobasidion annosum*; pust: *Punctularia strigosozonata*; disq: *Dichomitus squalens*; fome: *Fomitiporia mediterranea*; phca: *Phanerochaete carnosa*; sthi: *Stereum hirsutum*; auvu: *Auriscalpium vulgare*; aumi: *A. microsporum*; auor: *A. orientale*; phch: *Phanerochaete chrysosporium*; auor: *A. orientale*; stst: *Strobilurus stephanocystis*; stlu: *S. luchuensis*; stpa: *S. pachycystidiatusi*; stor: *S. orientalis.*

**Table S5.** Statistical analyses revealed that the average number of carbohydrate-active enzymes (CAZymes) in *Auriscalpium* and *Strobilurus* fungi.

| gene | auvu | aumi | auor | stst | stlu | stpa | stor | *Auriscalpium*_mean | *Strobilurus*_mean | log2(fc) | PValue |
|---|---|---|---|---|---|---|---|---|---|---|---|
| GH15 | 10 | 8 | 11 | 3 | 3 | 3 | 3 | 9.666667 | 3 | -1.6881 | 0.017054 |
| PL8 | 2 | 2 | 2 | 1 | 0 | 1 | 1 | 2 | 0.75 | -1.415 | 0.015392 |
| GH2 | 5 | 4 | 6 | 3 | 2 | 2 | 2 | 5 | 2.25 | -1.152 | 0.026338 |
| GH109 | 3 | 4 | 4 | 1 | 1 | 3 | 2 | 3.666667 | 1.75 | -1.0671 | 0.022544 |
| AA2 | 11 | 10 | 8 | 5 | 6 | 4 | 8 | 9.666667 | 5.75 | -0.7495 | 0.026183 |
| GH31 | 8 | 7 | 6 | 4 | 4 | 4 | 5 | 7 | 4.25 | -0.7199 | 0.026338 |
| GH3 | 13 | 12 | 10 | 7 | 7 | 7 | 11 | 11.66667 | 8 | -0.5443 | 0.040563 |
| GT4 | 4 | 4 | 4 | 6 | 5 | 5 | 5 | 4 | 5.25 | 0.3923 | 0.015392 |

| | | | | | | | | | | | |
|---|---|---|---|---|---|---|---|---|---|---|---|
| GT8 | 5 | 5 | 6 | 7 | 8 | 7 | 6 | 5.333333 | 7 | 0.3923 | 0.025031 |
| GH5 | 18 | 19 | 19 | 26 | 25 | 27 | 28 | 18.66667 | 26.5 | 0.5055 | 0.000266 |
| GH18 | 14 | 13 | 13 | 18 | 18 | 20 | 21 | 13.33333 | 19.25 | 0.5298 | 0.001846 |
| GH16 | 23 | 21 | 21 | 32 | 32 | 33 | 33 | 21.66667 | 32.5 | 0.585 | 0.001023 |
| GH12 | 2 | 2 | 2 | 3 | 3 | 3 | 4 | 2 | 3.25 | 0.7004 | 0.015392 |
| GH13 | 7 | 10 | 7 | 13 | 14 | 14 | 14 | 8 | 13.75 | 0.7814 | 0.023359 |
| AA5_1 | 6 | 5 | 5 | 11 | 10 | 9 | 10 | 5.333333 | 10 | 0.9069 | 0.000305 |
| GH55 | 2 | 2 | 2 | 3 | 4 | 4 | 4 | 2 | 3.75 | 0.9069 | 0.005986 |
| GT15 | 3 | 4 | 4 | 7 | 7 | 7 | 7 | 3.666667 | 7 | 0.9329 | 0.009852 |
| GH128 | 3 | 3 | 5 | 7 | 7 | 7 | 7 | 3.666667 | 7 | 0.9329 | 0.03775 |
| GH71 | 3 | 3 | 3 | 5 | 5 | 7 | 6 | 3 | 5.75 | 0.9386 | 0.010477 |
| CE8 | 2 | 3 | 3 | 6 | 5 | 5 | 5 | 2.666667 | 5.25 | 0.9773 | 0.003351 |
| GH27 | 3 | 2 | 3 | 5 | 6 | 5 | 5 | 2.666667 | 5.25 | 0.9773 | 0.003351 |
| CE4 | 5 | 6 | 6 | 9 | 10 | 13 | 14 | 5.666667 | 11.5 | 1.0211 | 0.01301 |
| GT33 | 1 | 1 | 1 | 2 | 2 | 2 | 3 | 1 | 2.25 | 1.1699 | 0.015392 |
| CE16 | 6 | 7 | 6 | 18 | 14 | 15 | 15 | 6.333333 | 15.5 | 1.2912 | 0.000731 |

| | | | | | | | | | | |
|---|---|---|---|---|---|---|---|---|---|---|
| GT1 | 2 | 3 | 2 | 6 | 6 | 6 | 5 | 2.333333 | 5.75 | 1.3012 | 0.001164 |
| GH105 | 2 | 3 | 4 | 7 | 7 | 8 | 8 | 3 | 7.5 | 1.3219 | 0.006057 |
| GH28 | 3 | 2 | 3 | 7 | 7 | 7 | 9 | 2.666667 | 7.5 | 1.4919 | 0.000565 |
| GH76 | 1 | 1 | 1 | 3 | 2 | 4 | 4 | 1 | 3.25 | 1.7004 | 0.01822 |
| CBM18 | 1 | 1 | 1 | 2 | 4 | 4 | 4 | 1 | 3.5 | 1.8074 | 0.015392 |
| GH43 | 3 | 3 | 4 | 10 | 14 | 14 | 13 | 3.333333 | 12.75 | 1.9355 | 0.001029 |
| GH17 | 1 | 1 | 1 | 3 | 4 | 4 | 5 | 1 | 4 | 2 | 0.005208 |
| GH29 | 0 | 0 | 0 | 1 | 1 | 1 | 2 | 0 | 1.25 | 2.3219 | 0.015392 |
| GH53 | 0 | 0 | 0 | 1 | 1 | 1 | 2 | 0 | 1.25 | 2.3219 | 0.015392 |
| GH93 | 0 | 0 | 0 | 1 | 1 | 2 | 2 | 0 | 1.5 | 2.585 | 0.013847 |
| GT17 | 0 | 0 | 0 | 1 | 2 | 2 | 1 | 0 | 1.5 | 2.585 | 0.013847 |
| GH35 | 1 | 1 | 1 | 4 | 6 | 5 | 9 | 1 | 6 | 2.585 | 0.018986 |
| GH135 | 0 | 0 | 0 | 3 | 1 | 2 | 2 | 0 | 2 | 3 | 0.016277 |
| PL4 | 0 | 0 | 0 | 2 | 1 | 2 | 3 | 0 | 2 | 3 | 0.016277 |
| GH45 | 0 | 0 | 0 | 1 | 1 | 3 | 3 | 0 | 2 | 3 | 0.040519 |
| GH127 | 0 | 0 | 0 | 2 | 2 | 1 | 4 | 0 | 2.25 | 3.1699 | 0.037386 |

| | | | | | | | | | | | |
|---|---|---|---|---|---|---|---|---|---|---|---|
| PL1 | 0 | 0 | 1 | 3 | 3 | 4 | 6 | 0.333333 | 4 | 3.585 | 0.008459 |
| CE12 | 0 | 0 | 0 | 3 | 2 | 5 | 6 | 0 | 4 | 4 | 0.022006 |
| CBM67 | 0 | 0 | 0 | 3 | 4 | 5 | 5 | 0 | 4.25 | 4.0875 | 0.003013 |

Note: auvu: *Auriscalpium vulgare*; aumi: *A. microsporum*; auor: *A. orientale*; stst: *Strobilurus stephanocystis*; stlu: *S. luchuensis*; stpa: *S. pachycystidiatusi*; stor: *S. orientalis.*

**Table S6.** The comparison of carbohydrate-active enzymes (CAZymes) between *Auriscalpium* and other WR fungi.

| gene | auvu | aumi | auor | phca | phch | hean | sthi | gama | disq | trve | fome | pust | *Auriscalpium* _mean | other white rot fungi_mean | log2(fc) | PValue |
|---|---|---|---|---|---|---|---|---|---|---|---|---|---|---|---|---|
| CBM60 | 3 | 4 | 3 | 0 | 0 | 0 | 1 | 6 | 0 | 0 | 0 | 0 | 3.333333 | 0.777778 | -2.0995 | 0.006249 |
| GH11 | 5 | 5 | 7 | 1 | 1 | 0 | 1 | 8 | 0 | 0 | 0 | 1 | 5.666667 | 1.333333 | -2.0875 | 0.003602 |
| GH15 | 10 | 8 | 11 | 2 | 2 | 4 | 3 | 3 | 2 | 4 | 1 | 4 | 9.666667 | 2.777778 | -1.7991 | 0.007537 |
| GH88 | 2 | 2 | 2 | 1 | 1 | 1 | 1 | 1 | 1 | 1 | 2 | 1 | 2 | 1.111111 | -0.848 | 4.37E-05 |
| CBM21 | 3 | 3 | 3 | 2 | 2 | 2 | 1 | 1 | 2 | 2 | 2 | 3 | 3 | 1.888889 | -0.6674 | 0.000543 |
| CBM50 | 8 | 7 | 8 | 5 | 7 | 5 | 4 | 5 | 4 | 6 | 6 | 6 | 7.666667 | 5.333333 | -0.5236 | 0.002145 |
| GH16 | 23 | 21 | 21 | 24 | 22 | 21 | 26 | 23 | 34 | 29 | 28 | 26 | 21.66667 | 25.88889 | 0.2569 | 0.018568 |

| | | | | | | | | | | | | | | | | |
|---|---|---|---|---|---|---|---|---|---|---|---|---|---|---|---|---|
| GH131 | 2 | 2 | 2 | 2 | 3 | 2 | 3 | 4 | 3 | 3 | 2 | 2 | 2 | 2.666667 | 0.415 | 0.022204 |
| CE16 | 6 | 7 | 6 | 5 | 7 | 7 | 11 | 10 | 13 | 8 | 6 | 11 | 6.333333 | 8.666667 | 0.4525 | 0.035898 |
| CBM48 | 1 | 1 | 1 | 2 | 2 | 1 | 1 | 1 | 2 | 1 | 1 | 2 | 1 | 1.444444 | 0.5305 | 0.035265 |
| GT69 | 2 | 2 | 2 | 3 | 3 | 4 | 3 | 2 | 4 | 3 | 2 | 3 | 2 | 3 | 0.585 | 0.002827 |
| CE15 | 1 | 1 | 1 | 3 | 2 | 1 | 1 | 1 | 2 | 2 | 1 | 2 | 1 | 1.666667 | 0.737 | 0.022204 |
| GH12 | 2 | 2 | 2 | 3 | 2 | 4 | 5 | 4 | 3 | 5 | 3 | 2 | 2 | 3.444444 | 0.7843 | 0.004993 |
| GH27 | 3 | 2 | 3 | 3 | 3 | 4 | 5 | 8 | 6 | 4 | 4 | 5 | 2.666667 | 4.666667 | 0.8074 | 0.009918 |
| AA2 | 11 | 10 | 8 | 12 | 17 | 13 | 8 | 32 | 14 | 27 | 18 | 13 | 9.666667 | 17.11111 | 0.8238 | 0.021584 |
| GH79 | 5 | 5 | 6 | 4 | 7 | 8 | 11 | 16 | 12 | 11 | 7 | 9 | 5.333333 | 9.444444 | 0.8244 | 0.007922 |
| GH43 | 3 | 3 | 4 | 4 | 4 | 6 | 11 | 10 | 7 | 3 | 6 | 9 | 3.333333 | 6.666667 | 1 | 0.008089 |
| GH20 | 2 | 2 | 2 | 3 | 4 | 4 | 4 | 2 | 4 | 5 | 6 | 6 | 2 | 4.222222 | 1.078 | 0.000906 |
| GH74 | 1 | 1 | 1 | 2 | 4 | 1 | 2 | 2 | 1 | 1 | 4 | 2 | 1 | 2.111111 | 1.078 | 0.021244 |
| GT1 | 2 | 3 | 2 | 8 | 6 | 2 | 5 | 13 | 8 | 20 | 10 | 12 | 2.333333 | 9.333333 | 2 | 0.003906 |
| GH28 | 3 | 2 | 3 | 4 | 5 | 8 | 17 | 18 | 7 | 10 | 16 | 13 | 2.666667 | 10.88889 | 2.0297 | 0.001541 |
| GH35 | 1 | 1 | 1 | 4 | 3 | 4 | 7 | 8 | 3 | 2 | 2 | 4 | 1 | 4.111111 | 2.0395 | 0.002085 |
| GT17 | 0 | 0 | 0 | 0 | 1 | 1 | 1 | 1 | 1 | 1 | 2 | 0 | 0 | 0.888889 | 3 | 0.002175 |

| | | | | | | | | | | | | | | | | |
|---|---|---|---|---|---|---|---|---|---|---|---|---|---|---|---|---|
| GH135 | 0 | 0 | 0 | 1 | 1 | 0 | 1 | 1 | 2 | 2 | 0 | 0 | 0 | 0.888889 | 3 | 0.009207 |
| GH32 | 0 | 0 | 0 | 0 | 0 | 1 | 1 | 1 | 2 | 3 | 0 | 1 | 0 | 1 | 3.1699 | 0.017072 |
| PL4 | 0 | 0 | 0 | 0 | 0 | 1 | 3 | 0 | 1 | 1 | 0 | 3 | 0 | 1 | 3.1699 | 0.039969 |
| GH53 | 0 | 0 | 0 | 1 | 1 | 1 | 2 | 3 | 1 | 1 | 1 | 2 | 0 | 1.444444 | 3.7004 | 0.000336 |
| GH127 | 0 | 0 | 0 | 1 | 1 | 1 | 1 | 2 | 1 | 3 | 1 | 3 | 0 | 1.555556 | 3.8074 | 0.000736 |
| CE12 | 0 | 0 | 0 | 0 | 0 | 2 | 3 | 4 | 2 | 0 | 2 | 1 | 0 | 1.555556 | 3.8074 | 0.011236 |
| CE1 | 0 | 0 | 0 | 2 | 4 | 2 | 1 | 2 | 0 | 3 | 0 | 2 | 0 | 1.777778 | 4 | 0.003451 |
| GH78 | 0 | 0 | 0 | 1 | 1 | 2 | 3 | 2 | 3 | 3 | 2 | 7 | 0 | 2.666667 | 4.585 | 0.002175 |

Note: auvu: *Auriscalpium vulgare*; aumi: *A. microsporum*; auor: *A. orientale*; phca: *Phanerochaete carnosa*; phch: *Phanerochaete chrysosporium*; hean: *Heterobasidion annosum*; sthi: *Stereum hirsutum*; gama: *Galerina marginata*; disq: *Dichomitus squalens*; trve: *Trametes versicolor*; fome: *Fomitiporia mediterranea*; pust: *Punctularia strigosozonata*.

**Table S7.** The comparison of carbohydrate-active enzymes (CAZymes) between *Strobilurus* and other WR fungi.

| gene | stst | stlu | stpa | stor | phca | phch | hean | sthi | gama | disq | trve | fome | pust | *Strobilurus*_mean | other white rot fungi_mean | log2(fc) | PValue |
|---|---|---|---|---|---|---|---|---|---|---|---|---|---|---|---|---|---|

| | | | | | | | | | | | | | | | | |
|---|---|---|---|---|---|---|---|---|---|---|---|---|---|---|---|---|
| GH62 | 1 | 1 | 1 | 1 | 0 | 0 | 0 | 0 | 1 | 0 | 0 | 0 | 0 | 1 | 0.111111111 | -3.1699 | 4.37E-05 |
| GT25 | 2 | 2 | 3 | 3 | 0 | 1 | 1 | 1 | 1 | 0 | 0 | 0 | 1 | 2.5 | 0.555555556 | -2.1699 | 0.001767 |
| CBM18 | 2 | 4 | 4 | 4 | 0 | 0 | 1 | 1 | 2 | 1 | 1 | 0 | 1 | 3.5 | 0.777777778 | -2.1699 | 0.006542 |
| GH93 | 1 | 1 | 2 | 2 | 0 | 0 | 0 | 1 | 0 | 1 | 0 | 0 | 1 | 1.5 | 0.333333333 | -2.1699 | 0.016625 |
| CBM67 | 3 | 4 | 5 | 5 | 0 | 0 | 0 | 0 | 9 | 0 | 0 | 0 | 0 | 4.25 | 1 | -2.0875 | 0.014156 |
| CBM60 | 3 | 4 | 2 | 3 | 0 | 0 | 0 | 1 | 6 | 0 | 0 | 0 | 0 | 3 | 0.777777778 | -1.9475 | 0.015597 |
| GH6 | 4 | 4 | 4 | 4 | 1 | 1 | 1 | 1 | 3 | 1 | 1 | 2 | 1 | 4 | 1.333333333 | -1.585 | 3.35E-06 |
| GT90 | 6 | 6 | 6 | 6 | 1 | 1 | 1 | 1 | 3 | 1 | 1 | 4 | 5 | 6 | 2 | -1.585 | 6.37E-05 |
| GH11 | 4 | 5 | 3 | 4 | 1 | 1 | 0 | 1 | 8 | 0 | 0 | 0 | 1 | 4 | 1.333333333 | -1.585 | 0.016955 |
| GH76 | 3 | 2 | 4 | 4 | 0 | 0 | 1 | 4 | 2 | 1 | 0 | 0 | 3 | 3.25 | 1.222222222 | -1.4109 | 0.016317 |
| GH17 | 3 | 4 | 4 | 5 | 1 | 1 | 1 | 1 | 4 | 1 | 1 | 4 | 1 | 4 | 1.666666667 | -1.263 | 0.003474 |
| GT33 | 2 | 2 | 2 | 3 | 1 | 1 | 1 | 1 | 1 | 1 | 1 | 1 | 1 | 2.25 | 1 | -1.1699 | 0.015392 |
| CBM21 | 4 | 4 | 4 | 4 | 2 | 2 | 2 | 1 | 1 | 2 | 2 | 2 | 3 | 4 | 1.888888889 | -1.0825 | 5.73E-06 |
| GH43 | 10 | 14 | 14 | 13 | 4 | 4 | 6 | 11 | 10 | 7 | 3 | 6 | 9 | 12.75 | 6.666666667 | -0.9355 | 0.001506 |
| GH55 | 3 | 4 | 4 | 4 | 2 | 2 | 1 | 2 | 3 | 2 | 2 | 1 | 3 | 3.75 | 2 | -0.9069 | 0.00085 |
| GH88 | 2 | 2 | 2 | 2 | 1 | 1 | 1 | 1 | 1 | 1 | 1 | 2 | 1 | 2 | 1.111111111 | -0.848 | 4.37E-05 |

| | | | | | | | | | | | | | | | | |
|---|---|---|---|---|---|---|---|---|---|---|---|---|---|---|---|---|
| CE16 | 18 | 14 | 15 | 15 | 5 | 7 | 7 | 11 | 10 | 13 | 8 | 6 | 11 | 15.5 | 8.666666667 | -0.8387 | 0.000389 |
| GH128 | 7 | 7 | 7 | 7 | 4 | 4 | 1 | 6 | 6 | 5 | 4 | 3 | 3 | 7 | 4 | -0.8074 | 0.000459 |
| CE8 | 6 | 5 | 5 | 5 | 2 | 2 | 3 | 4 | 3 | 3 | 2 | 3 | 6 | 5.25 | 3.111111111 | -0.7549 | 0.001154 |
| GH105 | 7 | 7 | 8 | 8 | 4 | 4 | 5 | 4 | 6 | 4 | 3 | 4 | 7 | 7.5 | 4.555555556 | -0.7193 | 0.000118 |
| AA7 | 22 | 27 | 24 | 31 | 20 | 10 | 18 | 15 | 30 | 11 | 9 | 12 | 19 | 26 | 16 | -0.7004 | 0.007291 |
| GT15 | 7 | 7 | 7 | 7 | 4 | 2 | 3 | 4 | 7 | 6 | 5 | 5 | 4 | 7 | 4.444444444 | -0.6554 | 0.000953 |
| CE4 | 9 | 10 | 13 | 14 | 4 | 6 | 7 | 9 | 14 | 7 | 5 | 7 | 7 | 11.5 | 7.333333333 | -0.6491 | 0.029298 |
| GH13 | 13 | 14 | 14 | 14 | 10 | 9 | 8 | 14 | 9 | 8 | 7 | 6 | 10 | 13.75 | 9 | -0.6114 | 0.000181 |
| GH71 | 5 | 5 | 7 | 6 | 2 | 3 | 3 | 8 | 3 | 4 | 5 | 2 | 5 | 5.75 | 3.888888889 | -0.5642 | 0.03979 |
| GH47 | 8 | 8 | 8 | 8 | 5 | 5 | 6 | 6 | 10 | 5 | 5 | 5 | 5 | 8 | 5.777777778 | -0.4695 | 0.003626 |
| GT69 | 4 | 4 | 4 | 4 | 3 | 3 | 4 | 3 | 2 | 4 | 3 | 2 | 3 | 4 | 3 | -0.415 | 0.002827 |
| GT8 | 7 | 8 | 7 | 6 | 7 | 7 | 6 | 7 | 4 | 6 | 5 | 4 | 4 | 7 | 5.555555556 | -0.3334 | 0.039263 |
| GH16 | 32 | 32 | 33 | 33 | 24 | 22 | 21 | 26 | 23 | 34 | 29 | 28 | 26 | 32.5 | 25.88888889 | -0.3281 | 0.001078 |
| GH5 | 26 | 25 | 27 | 28 | 23 | 19 | 16 | 20 | 37 | 20 | 22 | 19 | 17 | 26.5 | 21.44444444 | -0.3054 | 0.043942 |
| GT4 | 6 | 5 | 5 | 5 | 5 | 4 | 4 | 4 | 5 | 4 | 4 | 3 | 6 | 5.25 | 4.333333333 | -0.2768 | 0.037762 |
| GT22 | 3 | 3 | 3 | 3 | 5 | 4 | 3 | 4 | 3 | 4 | 4 | 3 | 3 | 3 | 3.666666667 | 0.2895 | 0.022204 |

| | | | | | | | | | | | | | | | | |
|---|---|---|---|---|---|---|---|---|---|---|---|---|---|---|---|---|
| GH131 | 2 | 2 | 2 | 2 | 2 | 3 | 2 | 3 | 4 | 3 | 3 | 2 | 2 | 2 | 2.666666667 | 0.415 | 0.022204 |
| PL14 | 3 | 3 | 4 | 4 | 5 | 5 | 4 | 4 | 4 | 7 | 6 | 4 | 4 | 3.5 | 4.777777778 | 0.449 | 0.019941 |
| GH3 | 7 | 7 | 7 | 11 | 11 | 10 | 12 | 17 | 11 | 8 | 12 | 8 | 13 | 8 | 11.33333333 | 0.5025 | 0.039207 |
| GH31 | 4 | 4 | 4 | 5 | 6 | 6 | 9 | 8 | 4 | 6 | 5 | 5 | 7 | 4.25 | 6.222222222 | 0.55 | 0.006105 |
| CE15 | 1 | 1 | 1 | 1 | 3 | 2 | 1 | 1 | 1 | 2 | 2 | 1 | 2 | 1 | 1.666666667 | 0.737 | 0.022204 |
| GH20 | 2 | 2 | 3 | 2 | 3 | 4 | 4 | 4 | 2 | 4 | 5 | 6 | 6 | 2.25 | 4.222222222 | 0.9081 | 0.002331 |
| GH74 | 1 | 1 | 1 | 1 | 2 | 4 | 1 | 2 | 2 | 1 | 1 | 4 | 2 | 1 | 2.111111111 | 1.078 | 0.021244 |
| GH109 | 1 | 1 | 3 | 2 | 4 | 4 | 3 | 4 | 5 | 3 | 5 | 2 | 5 | 1.75 | 3.888888889 | 1.152 | 0.010139 |
| GH78 | 1 | 1 | 1 | 1 | 1 | 1 | 2 | 3 | 2 | 3 | 3 | 2 | 7 | 1 | 2.666666667 | 1.415 | 0.024166 |
| GH79 | 4 | 3 | 4 | 3 | 4 | 7 | 8 | 11 | 16 | 12 | 11 | 7 | 9 | 3.5 | 9.444444444 | 1.4321 | 0.000823 |
| AA2 | 5 | 6 | 4 | 8 | 12 | 17 | 13 | 8 | 32 | 14 | 27 | 18 | 13 | 5.75 | 17.11111111 | 1.5733 | 0.002001 |
| CE1 | 0 | 0 | 1 | 1 | 2 | 4 | 2 | 1 | 2 | 0 | 3 | 0 | 2 | 0.5 | 1.777777778 | 1.8301 | 0.032259 |
| GT41 | 0 | 0 | 0 | 1 | 1 | 1 | 1 | 0 | 0 | 2 | 2 | 1 | 2 | 0.25 | 1.111111111 | 2.152 | 0.040764 |
| CE14 | 0 | 0 | 0 | 0 | 1 | 1 | 1 | 0 | 1 | 0 | 1 | 0 | 1 | 0 | 0.666666667 | 2.585 | 0.00395 |
| GH32 | 0 | 0 | 0 | 0 | 0 | 0 | 1 | 1 | 1 | 2 | 3 | 0 | 1 | 0 | 1 | 3.1699 | 0.017072 |

Note: stst: *Strobilurus stephanocystis*; stlu: *S. luchuensis*; stpa: *S. pachycystidiatusi*; stor: *S. orientalis.* phca: *Phanerochaete carnosa*; phch: *Phanerochaete chrysosporium*; hean: *Heterobasidion annosum*; sthi: *Stereum hirsutum*; gama: *Galerina marginata*; disq: *Dichomitus squalens*; trve: *Trametes versicolor*; fome: *Fomitiporia mediterranea*; pust: *Punctularia strigosozonata*.

**Table S8.** Gene distribution of lignocellulolytic genes in *Auriscalpium* and *Strobilurus* fungi and the other 15 fungi.

| gene | dapr | copu | sela | woco | fopi | gltr | gama | trve | hean | pust | disq | fome | phch | phca | sthi | auvu | aumi | auor | stst | stlu | stpa | stor |
|---|---|---|---|---|---|---|---|---|---|---|---|---|---|---|---|---|---|---|---|---|---|---|
| all | 33 | 73 | 51 | 38 | 50 | 51 | 164 | 115 | 93 | 113 | 81 | 103 | 97 | 94 | 133 | 112 | 97 | 111 | 107 | 106 | 122 | 111 |
| AA9 | 0 | 10 | 5 | 1 | 4 | 1 | 15 | 16 | 8 | 12 | 16 | 12 | 14 | 11 | 13 | 11 | 10 | 14 | 14 | 13 | 15 | 16 |
| GH1 | 1 | 3 | 3 | 1 | 2 | 5 | 5 | 2 | 2 | 1 | 3 | 5 | 2 | 2 | 3 | 4 | 3 | 3 | 3 | 3 | 3 | 3 |
| GH12 | 1 | 4 | 2 | 2 | 2 | 2 | 4 | 5 | 4 | 2 | 3 | 3 | 2 | 3 | 5 | 2 | 2 | 1 | 3 | 3 | 3 | 4 |
| GH3 | 7 | 10 | 8 | 6 | 9 | 8 | 9 | 9 | 9 | 12 | 5 | 7 | 8 | 9 | 13 | 9 | 9 | 7 | 6 | 6 | 6 | 9 |
| GH44 | 0 | 0 | 0 | 0 | 0 | 0 | 2 | 0 | 0 | 1 | 1 | 0 | 0 | 0 | 1 | 0 | 0 | 0 | 1 | 1 | 1 | 1 |
| GH45 | 0 | 0 | 0 | 0 | 0 | 0 | 0 | 1 | 1 | 0 | 1 | 0 | 0 | 0 | 0 | 0 | 0 | 0 | 1 | 1 | 3 | 3 |
| GH5_5 | 3 | 5 | 3 | 2 | 3 | 2 | 7 | 3 | 3 | 3 | 1 | 3 | 2 | 6 | 4 | 2 | 2 | 2 | 8 | 7 | 7 | 4 |
| GH6 | 0 | 1 | 1 | 0 | 0 | 0 | 3 | 1 | 1 | 1 | 1 | 2 | 1 | 1 | 1 | 1 | 1 | 1 | 2 | 2 | 2 | 4 |
| GH7 | 0 | 2 | 0 | 0 | 0 | 0 | 6 | 4 | 1 | 5 | 0 | 2 | 8 | 5 | 2 | 4 | 5 | 4 | 2 | 1 | 2 | 3 |
| GH74 | 0 | 0 | 0 | 0 | 0 | 0 | 0 | 0 | 0 | 0 | 0 | 0 | 0 | 0 | 0 | 0 | 0 | 0 | 0 | 0 | 0 | 1 |

| | | | | | | | | | | | | | | | | | | | | | | |
|---|---|---|---|---|---|---|---|---|---|---|---|---|---|---|---|---|---|---|---|---|---|---|
| GH9 | 1 | 1 | 1 | 0 | 0 | 1 | 1 | 1 | 1 | 1 | 1 | 1 | 1 | 1 | 1 | 1 | 1 | 1 | 1 | 1 | 1 | 1 |
| CE1 | 0 | 0 | 0 | 0 | 0 | 1 | 2 | 3 | 1 | 2 | 0 | 0 | 4 | 2 | 1 | 0 | 0 | 0 | 0 | 0 | 1 | 0 |
| CE5 | 0 | 0 | 0 | 0 | 0 | 0 | 4 | 0 | 0 | 1 | 0 | 0 | 0 | 0 | 1 | 0 | 0 | 0 | 0 | 0 | 0 | 0 |
| GH10 | 3 | 3 | 1 | 4 | 3 | 3 | 9 | 6 | 2 | 5 | 5 | 2 | 6 | 4 | 6 | 6 | 5 | 6 | 3 | 4 | 6 | 4 |
| GH11 | 0 | 0 | 0 | 0 | 0 | 0 | 8 | 0 | 0 | 1 | 0 | 0 | 1 | 1 | 1 | 5 | 5 | 7 | 4 | 5 | 3 | 4 |
| GH3 | 1 | 2 | 1 | 1 | 2 | 1 | 1 | 2 | 2 | 0 | 2 | 0 | 1 | 1 | 3 | 3 | 2 | 2 | 0 | 0 | 0 | 1 |
| GH43_13 | 0 | 0 | 0 | 0 | 0 | 0 | 0 | 0 | 0 | 0 | 0 | 0 | 0 | 0 | 0 | 0 | 0 | 0 | 0 | 0 | 0 | 1 |
| GH43_30 | 0 | 0 | 0 | 0 | 0 | 0 | 0 | 0 | 0 | 0 | 1 | 0 | 0 | 0 | 0 | 0 | 0 | 0 | 0 | 0 | 0 | 0 |
| CE8 | 3 | 2 | 2 | 1 | 2 | 2 | 3 | 2 | 3 | 6 | 3 | 3 | 2 | 2 | 4 | 2 | 3 | 3 | 6 | 5 | 5 | 5 |
| GH28 | 0 | 3 | 1 | 2 | 2 | 1 | 1 | 1 | 1 | 3 | 1 | 3 | 1 | 0 | 4 | 1 | 0 | 0 | 1 | 1 | 0 | 3 |
| PL1 | 0 | 0 | 0 | 0 | 0 | 0 | 0 | 0 | 0 | 2 | 0 | 0 | 0 | 0 | 0 | 0 | 0 | 0 | 0 | 0 | 0 | 0 |
| PL1_2 | 0 | 0 | 0 | 0 | 0 | 0 | 0 | 0 | 0 | 1 | 0 | 0 | 0 | 0 | 0 | 0 | 0 | 0 | 0 | 0 | 0 | 1 |
| PL1_4 | 0 | 0 | 0 | 0 | 0 | 0 | 0 | 0 | 1 | 1 | 0 | 0 | 0 | 0 | 2 | 0 | 0 | 0 | 2 | 2 | 3 | 3 |
| PL1_7 | 0 | 0 | 0 | 0 | 0 | 0 | 2 | 0 | 1 | 0 | 0 | 2 | 0 | 0 | 2 | 0 | 0 | 0 | 0 | 0 | 1 | 0 |
| AA1 | 3 | 1 | 1 | 1 | 1 | 0 | 0 | 1 | 3 | 0 | 1 | 0 | 4 | 9 | 3 | 1 | 1 | 1 | 3 | 3 | 3 | 2 |
| AA1_1 | 0 | 6 | 4 | 3 | 5 | 4 | 8 | 7 | 14 | 12 | 11 | 10 | 0 | 0 | 16 | 10 | 8 | 13 | 8 | 8 | 12 | 10 |

| | | | | | | | | | | | | | | | | | | | | | | |
|---|---|---|---|---|---|---|---|---|---|---|---|---|---|---|---|---|---|---|---|---|---|---|
| AA1_2 | 2 | 1 | 1 | 1 | 1 | 1 | 1 | 2 | 1 | 1 | 1 | 1 | 1 | 1 | 2 | 1 | 1 | 1 | 1 | 2 | 3 | 4 |
| AA2 | 1 | 1 | 1 | 2 | 2 | 1 | 24 | 26 | 9 | 12 | 14 | 18 | 17 | 12 | 7 | 10 | 10 | 8 | 4 | 4 | 4 | 8 |
| AA3_1 | 0 | 4 | 5 | 0 | 0 | 1 | 1 | 3 | 2 | 1 | 0 | 1 | 2 | 2 | 2 | 2 | 1 | 2 | 2 | 2 | 1 | 0 |
| AA3_2a | 0 | 1 | 0 | 0 | 1 | 5 | 19 | 3 | 12 | 9 | 0 | 2 | 3 | 6 | 15 | 24 | 15 | 22 | 12 | 11 | 16 | 3 |
| AA3_2b | 0 | 0 | 0 | 0 | 1 | 1 | 3 | 2 | 1 | 0 | 0 | 16 | 2 | 3 | 5 | 0 | 0 | 0 | 1 | 1 | 1 | 1 |
| AA3_3 | 1 | 5 | 6 | 6 | 5 | 2 | 6 | 4 | 3 | 5 | 0 | 3 | 3 | 4 | 7 | 6 | 7 | 5 | 6 | 8 | 9 | 0 |
| AA3_4 | 0 | 0 | 0 | 0 | 0 | 1 | 0 | 1 | 0 | 1 | 0 | 0 | 1 | 0 | 0 | 0 | 0 | 0 | 0 | 0 | 0 | 0 |
| AA4 | 2 | 0 | 0 | 0 | 0 | 3 | 0 | 0 | 0 | 0 | 0 | 0 | 0 | 0 | 0 | 0 | 0 | 0 | 0 | 0 | 0 | 0 |
| AA5_1 | 3 | 6 | 3 | 4 | 4 | 2 | 17 | 9 | 5 | 9 | 6 | 4 | 7 | 6 | 8 | 6 | 5 | 5 | 11 | 10 | 9 | 5 |
| AA5_2 | 0 | 0 | 0 | 0 | 0 | 0 | 0 | 0 | 0 | 0 | 3 | 0 | 0 | 0 | 0 | 0 | 0 | 0 | 0 | 0 | 0 | 5 |
| AA6 | 1 | 2 | 2 | 1 | 1 | 3 | 3 | 1 | 2 | 3 | 1 | 3 | 4 | 3 | 1 | 1 | 1 | 3 | 2 | 2 | 2 | 2 |

Note: dapr: *Dacryopinax primogenitus*; copu: *Coniophora puteana*; sela: *Serpula lacrymans* ; woco: *Wolfiporia cocos*; fopi: *Fomitopsis pinicola*i; gltr: *Gloeophyllum trabeum*; gama: *Galerina marginata*; trve: *Trametes versicolor*; hean: *Heterobasidion annosum*; pust: *Punctularia strigosozonata*; disq: *Dichomitus squalens*; fome: *Fomitiporia mediterranea*; phca: *Phanerochaete carnosa*; sthi: *Stereum hirsutum*; auvu: *Auriscalpium vulgare*; aumi: *A. microsporum*; auor: *A. orientale*; phch: *Phanerochaete chrysosporium*; auor: *A. orientale*; stst: *Strobilurus stephanocystis*; stlu: *S. luchuensis*; stpa: *S. pachycystidiatusi*; stor: *S. orientalis.*

**Table S9.** Statistical analyses revealed that the average number of lignocellulolytic genes in *Auriscalpium* and *Strobilurus* fungi.

| gene | auvu | aumi | auor | stst | stlu | stpa | stor | *Auriscalpium*_mean | *Strobilurus*_mean | log2(fc) | PValue |
|---|---|---|---|---|---|---|---|---|---|---|---|
| GH3 | 3 | 2 | 2 | 0 | 0 | 0 | 1 | 2.333333 | 0.25 | -3.2224 | 0.007335 |
| GH7 | 4 | 5 | 4 | 2 | 1 | 2 | 3 | 4.333333 | 2 | -1.1155 | 0.006846 |
| AA2 | 10 | 10 | 8 | 4 | 4 | 4 | 8 | 9.333333 | 5 | -0.9005 | 0.016409 |
| GH12 | 2 | 2 | 1 | 3 | 3 | 3 | 4 | 1.666667 | 3.25 | 0.9635 | 0.018824 |
| CE8 | 2 | 3 | 3 | 6 | 5 | 5 | 5 | 2.666667 | 5.25 | 0.9773 | 0.003351 |
| AA1 | 1 | 1 | 1 | 3 | 3 | 3 | 2 | 1 | 2.75 | 1.4594 | 0.005986 |
| GH5_5 | 2 | 2 | 2 | 8 | 7 | 7 | 4 | 2 | 6.5 | 1.7004 | 0.013847 |
| GH45 | 0 | 0 | 0 | 1 | 1 | 3 | 3 | 0 | 2 | 3 | 0.040519 |
| PL1_4 | 0 | 0 | 0 | 2 | 2 | 3 | 3 | 0 | 2.5 | 3.3219 | 0.003239 |

Note: auvu: *Auriscalpium vulgare*; aumi: *A. microsporum*; auor: *A. orientale*; stst: *Strobilurus stephanocystis*; stlu: *S. luchuensis*; stpa: *S. pachycystidiatusi*; stor: *S. orientalis.*

**Table S10.** Raw data of four major chemical components of cones before and after decomposition by fungi in *Auriscalpium*.

| cones | compositions | sample 1 | sample 2 | sample 3 | sample 4 | sample 5 | sample 6 | sample 7 | sample 8 | sample 9 | sample 10 | mean | variance |
|---|---|---|---|---|---|---|---|---|---|---|---|---|---|
| *P. yunnanensis* | Lignin(before) | 28.88 % | 27.70 % | 29.64 % | 27.39 % | 28.55 % | 26.16 % | 30.01 % | 28.20 % | 26.26 % | 27.91 % | 28.07% | 0.0128 |
| *P. yunnanensis* | Lignin(after) | 16.71 % | 17.86 % | 17.06 % | 14.57 % | 18.76 % | 17.53 % | 16.57 % | 20.28 % | 15.09 % | 19.26 % | 17.37% | 0.0178 |
| *P. yunnanensis* | cellulose(before) | 40.10 % | 38.39 % | 40.02 % | 39.74 % | 40.69 % | 39.98 % | 38.76 % | 40.91 % | 39.76 % | 39.50 % | 39.79% | 0.0077 |
| *P. yunnanensis* | cellulose(after) | 51.47 % | 53.68 % | 53.37 % | 53.62 % | 50.04 % | 52.99 % | 54.10 % | 52.47 % | 51.23 % | 55.54 % | 52.85% | 0.016 |
| *P. yunnanensis* | hemicellulose(before) | 20.84 % | 20.60 % | 21.86 % | 21.09 % | 20.45 % | 21.65 % | 20.39 % | 20.89 % | 21.31 % | 21.32 % | 21.04% | 0.005 |
| *P. yunnanensis* | hemicellulose(after) | 13.26 % | 14.17 % | 14.62 % | 15.59 % | 15.58 % | 14.38 % | 16.56 % | 13.41 % | 15.48 % | 13.47 % | 14.65% | 0.0112 |
| *P. yunnanensis* | pectin(before) | 2.12 % | 2.32 % | 2.74 % | 2.15 % | 2.29 % | 2.34 % | 2.25 % | 2.78 % | 2.43 % | 2.89 % | 2.43% | 0.0027 |
| *P. yunnanensis* | pectin(after) | 3.03 % | 3.10 % | 3.50 % | 3.12 % | 3.33 % | 3.95 % | 3.02 % | 3.85 % | 3.32 % | 3.97 % | 3.42% | 0.0038 |
| *P. sylvestris* | Lignin(before) | 27.27 % | 27.93 % | 27.02 % | 28.11 % | 27.9 % | 27.01 % | 27.29 % | 27.45 % | 28.19 % | 26.45 % | 27.46% | 0.0056 |
| *P. sylvestris* | Lignin(after) | 20.51 % | 19.51 % | 19.52 % | 20.89 % | 20.43 % | 21.31 % | 21.28 % | 23.25 % | 23.16 % | 22.46 % | 21.23% | 0.0135 |
| *P. sylvestris* | cellulose(before) | 42.61 % | 42.63 % | 41.89 % | 41.11 % | 43.70 % | 43.46 % | 42.89 % | 43.05 % | 41.47 % | 42.68 % | 42.55% | 0.0083 |
| *P. sylvestris* | cellulose(after) | 52.94 % | 50.71 % | 52.23 % | 52.48 % | 51.45 % | 51.04 % | 51.67 % | 52.02 % | 52.45 % | 50.47 % | 51.75% | 0.0082 |
| *P. sylvestris* | hemicellulose(before) | 19.52 % | 18.99 % | 19.32 % | 19.1 % | 19.32 % | 18.40 % | 18.80 % | 19.03 % | 19.63 % | 19.24 % | 19.14% | 0.0036 |
| *P. sylvestris* | hemicellulose(after) | 14.6 % | 12.58 % | 13.86 % | 11.45 % | 11.37 % | 11.28 % | 11.01 % | 13.01 % | 13.75 % | 13.33 % | 12.62% | 0.0128 |

| | | | | | | | | | | | | | |
|---|---|---|---|---|---|---|---|---|---|---|---|---|---|
| *P. sylvestris* | pectin(before) | 2.12 % | 2.24 % | 2.12 % | 2.23 % | 2.65 % | 3.12 % | 2.16 % | 2.24 % | 2.24 % | 2.17 % | 2.33% | 0.0032 |
| *P. sylvestris* | pectin(after) | 3.28 % | 3.47 % | 3.83 % | 3.24 % | 3.33 % | 3.74 % | 3.55 % | 3.14 % | 3.75 % | 3.05 % | 3.44% | 0.0027 |
| *P. armandii* | Lignin(before) | 23.37 % | 22.98 % | 23.48 % | 22.89 % | 23.97 % | 23.72 % | 23.01 % | 22.84 % | 22.47 % | 23.66 % | 23.24% | 0.0047 |
| *P. armandii* | Lignin(after) | 18.29 % | 15.3 % | 16.67 % | 18.84 % | 17.66 % | 16.65 % | 17.52 % | 15.93 % | 15.42 % | 17.02 % | 16.93% | 0.0118 |
| *P. armandii* | cellulose(before) | 47.71 % | 46.64 % | 46.71 % | 46.59 % | 46.64 % | 46.36 % | 47.56 % | 47.45 % | 47.91 % | 46.68 % | 47.03% | 0.0056 |
| *P. armandii* | cellulose(after) | 53.57 % | 57.05 % | 56.68 % | 55.82 % | 53.79 % | 55.48 % | 55.98 % | 54.96 % | 57.50 % | 56.85 % | 55.77% | 0.0134 |
| *P. armandii* | hemicellulose(before) | 20.27 % | 20.38 % | 19.9 % | 20.49 % | 19.87 % | 20.05 % | 20.50 % | 20.97 % | 21.06 % | 19.94 % | 20.34% | 0.0043 |
| *P. armandii* | hemicellulose(after) | 13.81 % | 13.23 % | 13.21 % | 12.57 % | 13.63 % | 13.26 % | 12.21 % | 12.43 % | 12.99 % | 13.76 % | 13.11% | 0.0056 |
| *P. armandii* | pectin(before) | 2.89 % | 2.86 % | 2.32 % | 2.45 % | 2.39 % | 2.46 % | 2.78 % | 2.60 % | 2.52 % | 2.53 % | 2.58% | 0.002 |
| *P. armandii* | pectin(after) | 3.08 % | 3.25 % | 3.35 % | 3.28 % | 3.37 % | 3.04 % | 4.36 % | 3.35 % | 2.83 % | 3.67 % | 3.36% | 0.0042 |

**Table S11: Gene families expansion of CAZymes in 22 fungi.**

We uploaded it separately.
