# Supplementary figures and images for: Genomic and Experimental Investigations of Auriscalpium and Strobilurus Fungi Reveal New Insights into Pinecone Decomposition
Source: J Fungi (Basel). 2021 Aug 23;7(8):679. doi: 10.3390/jof7080679 (PMC8401616; doi:10.3390/jof7080679)

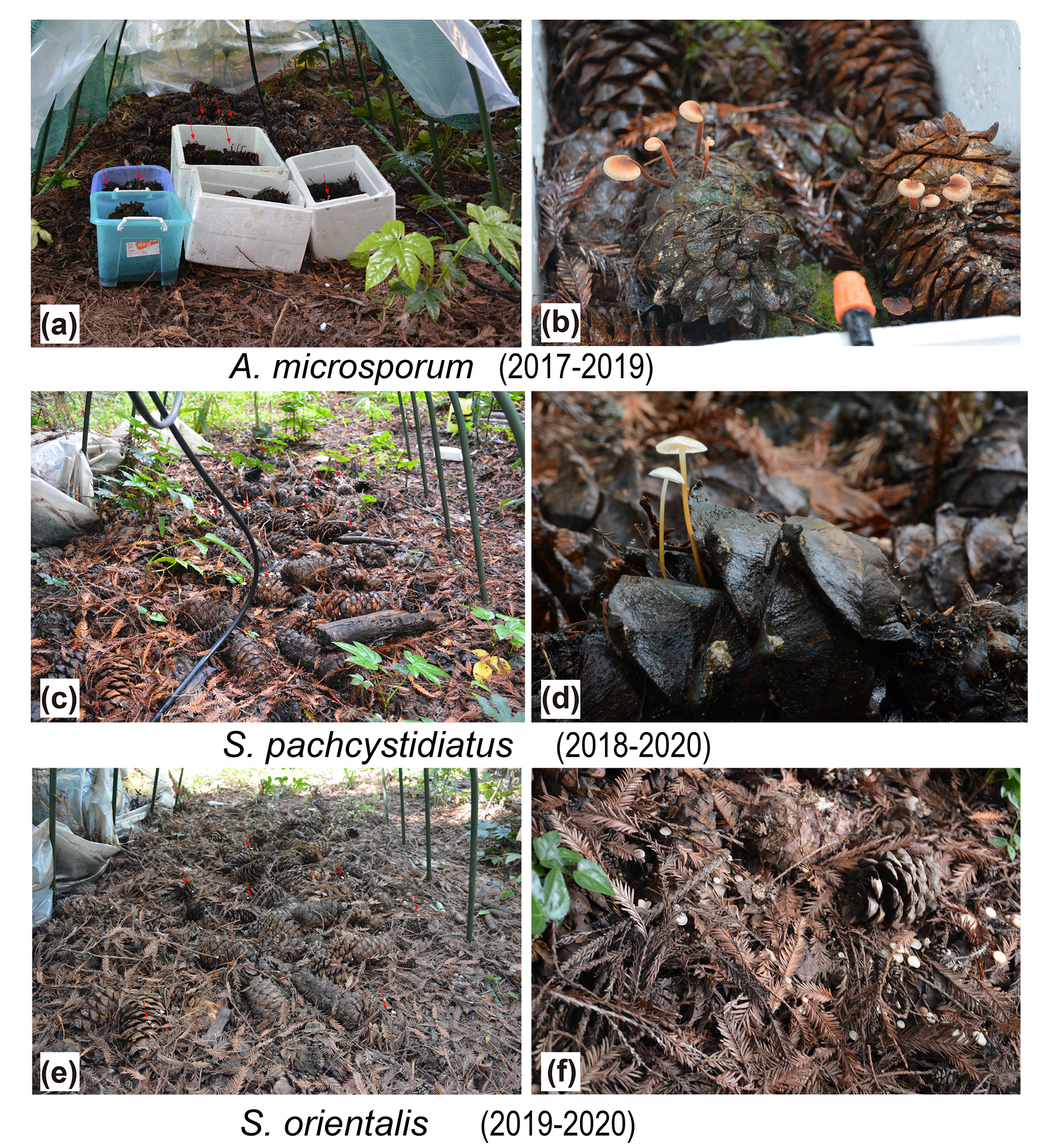

Supplement: Supplementary file 1 [file jof-07-00679-s001.zip › jof-1317844-supplementary/Supplementary Materials/Fig. S2.tif]

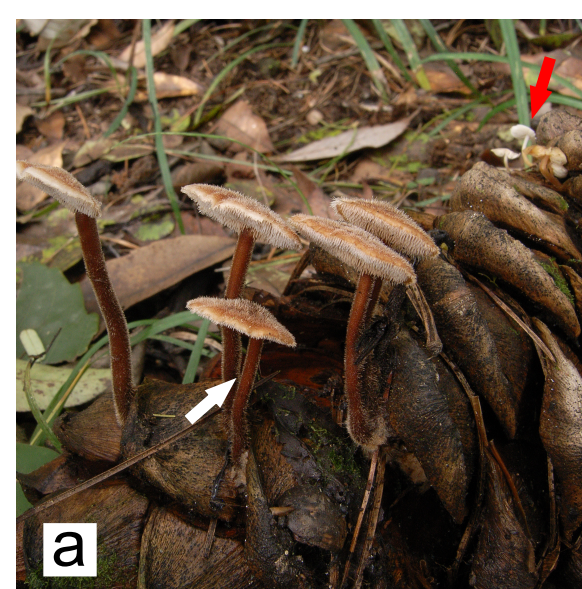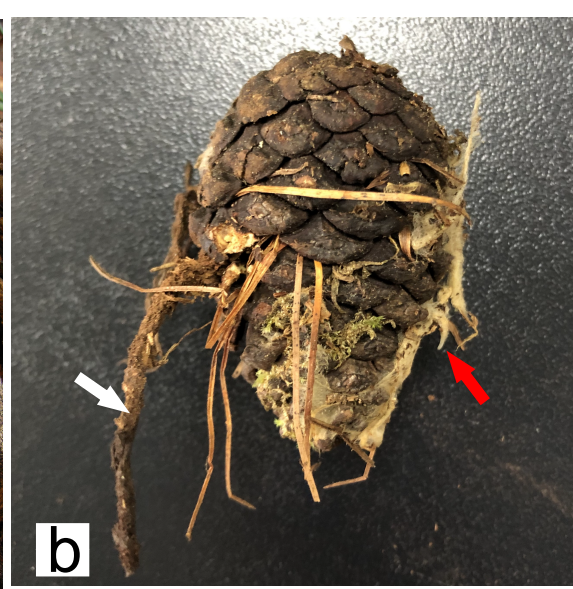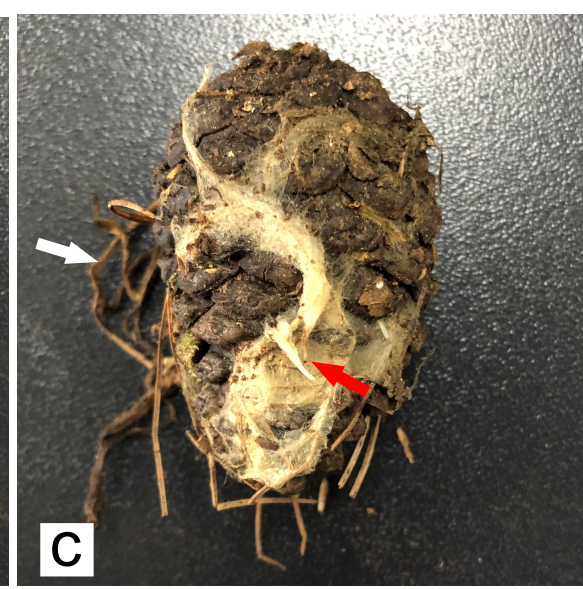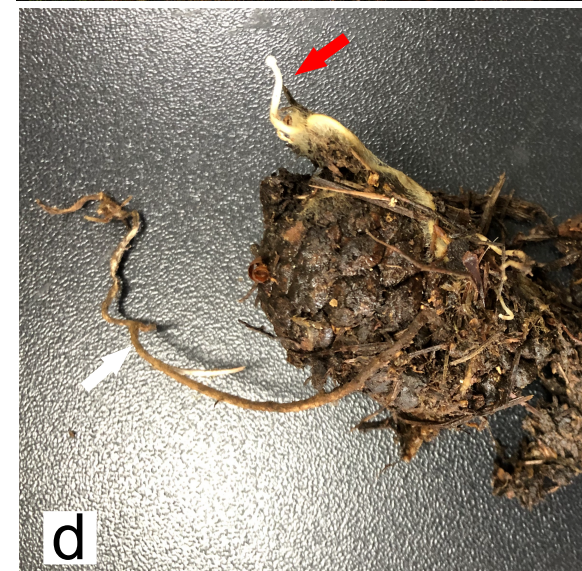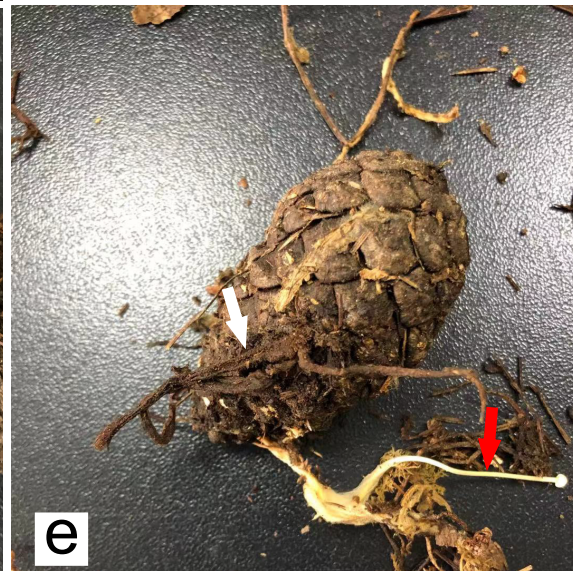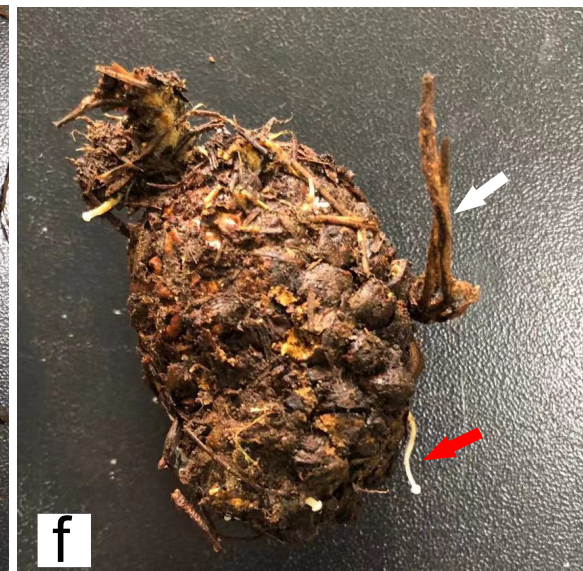

Supplement: Supplementary file 1 [file jof-07-00679-s001.zip › jof-1317844-supplementary/Supplementary Materials/Fig. S3.pdf]

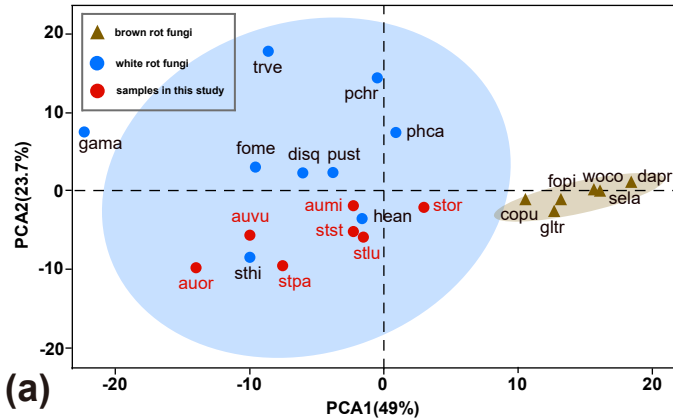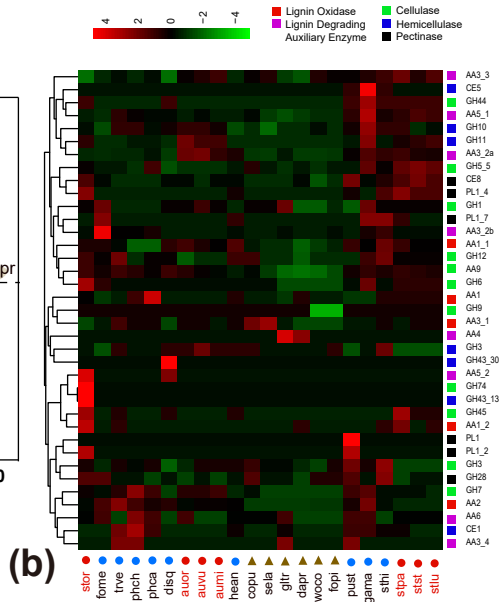

Supplement: Supplementary file 1 [file jof-07-00679-s001.zip › jof-1317844-supplementary/Supplementary Materials/Fig. S4.pdf]

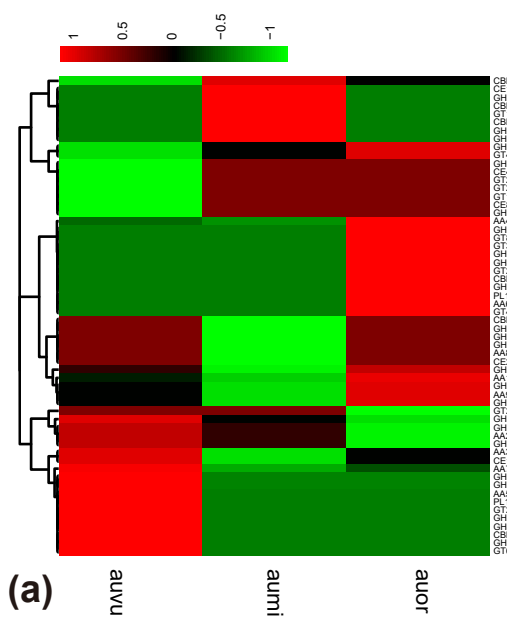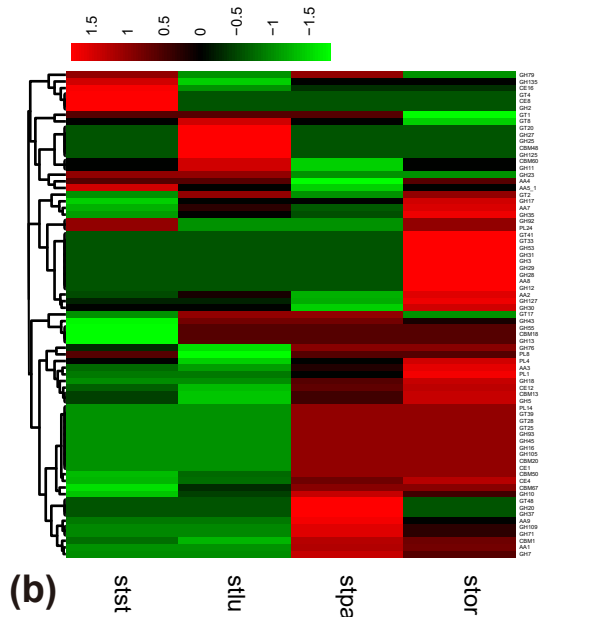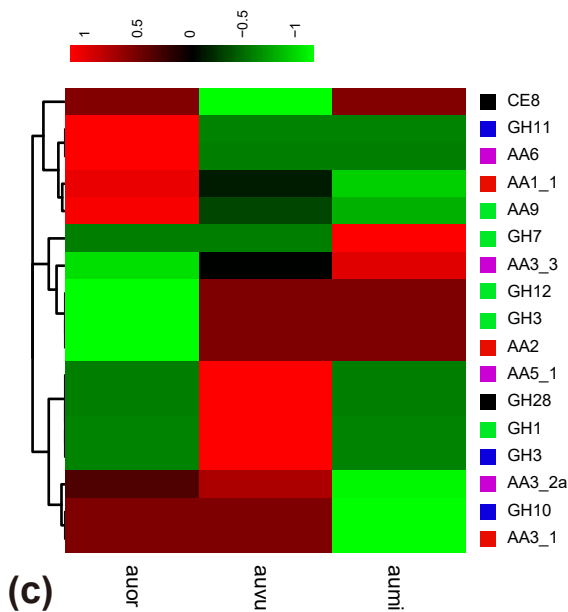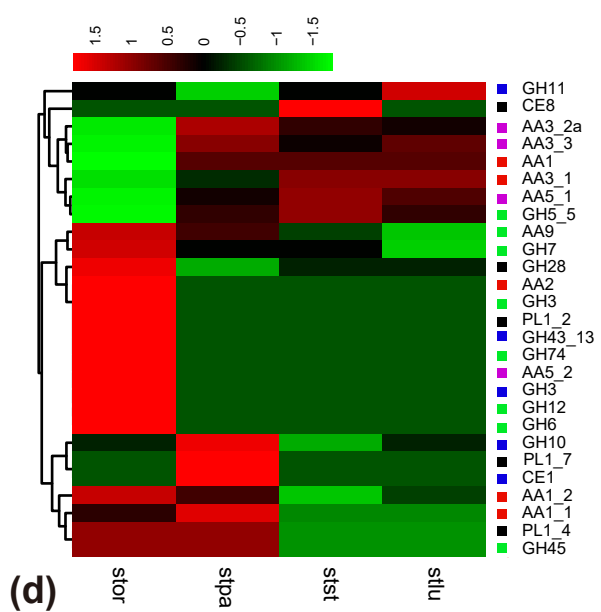

Supplement: Supplementary file 1 [file jof-07-00679-s001.zip › jof-1317844-supplementary/Supplementary Materials/Fig. S5.pdf]
